# The Effects of Context and Attention on Spiking Activity in Human Early Visual Cortex

**DOI:** 10.1371/journal.pbio.1002420

**Published:** 2016-03-25

**Authors:** Matthew W. Self, Judith C. Peters, Jessy K. Possel, Joel Reithler, Rainer Goebel, Peterjan Ris, Danique Jeurissen, Leila Reddy, Steven Claus, Johannes C. Baayen, Pieter R. Roelfsema

**Affiliations:** 1 Department of Vision and Cognition, Netherlands Institute for Neuroscience, an institute of the Royal Netherlands Academy of Art and Sciences (KNAW), Amsterdam, the Netherlands; 2 Cognitive Neuroscience Department, Faculty of Psychology and Neuroscience, Maastricht University, Maastricht, the Netherlands; 3 Department of Neuroimaging and Neuromodeling, Netherlands Institute for Neuroscience, an institute of the Royal Netherlands Academy of Art and Sciences (KNAW), Amsterdam, the Netherlands; 4 Department of Clinical Neurophysiology, VU University Medical Center, Amsterdam, the Netherlands; 5 Université de Toulouse, Centre de Recherche Cerveau et Cognition, Université Paul Sabatier, Toulouse, France; 6 CNRS, UMR 5549, Faculté de Médecine de Purpan, Toulouse, France; 7 Department of Clinical Neurophysiology, Stichting Epilepsie Instelling Nederland, Heemstede, the Netherlands; 8 Department of Neurosurgery, VU University Medical Center, Amsterdam, the Netherlands; 9 Department of Integrative Neurophysiology, Center for Neurogenomics and Cognitive Research, VU University, Amsterdam, The Netherlands; 10 Psychiatry department, Academic Medical Center, Amsterdam, The Netherlands; McGill University, CANADA

## Abstract

Here we report the first quantitative analysis of spiking activity in human early visual cortex. We recorded multi-unit activity from two electrodes in area V2/V3 of a human patient implanted with depth electrodes as part of her treatment for epilepsy. We observed well-localized multi-unit receptive fields with tunings for contrast, orientation, spatial frequency, and size, similar to those reported in the macaque. We also observed pronounced gamma oscillations in the local-field potential that could be used to estimate the underlying spiking response properties. Spiking responses were modulated by visual context and attention. We observed orientation-tuned surround suppression: responses were suppressed by image regions with a uniform orientation and enhanced by orientation contrast. Additionally, responses were enhanced on regions that perceptually segregated from the background, indicating that neurons in the human visual cortex are sensitive to figure-ground structure. Spiking responses were also modulated by object-based attention. When the patient mentally traced a curve through the neurons’ receptive fields, the accompanying shift of attention enhanced neuronal activity. These results demonstrate that the tuning properties of cells in the human early visual cortex are similar to those in the macaque and that responses can be modulated by both contextual factors and behavioral relevance. Our results, therefore, imply that the macaque visual system is an excellent model for the human visual cortex.

## Abstract 2

Quantitative analysis of spiking activity of neurons in area V2/V3 of the human visual cortex shows remarkable similarities to the response properties of neurons in the early visual cortex of the macaque monkey, justifying extrapolation between species.

## Author Summary

Our knowledge of the function of the early visual cortex is based largely on recordings of spiking activity from neurons in animal models, in particular the macaque monkey. Indirect measurements of neuronal activity in the human visual cortex have suggested many similarities with the macaque visual cortex, but to date there have been no quantitative analyses of spiking data in the human early visual cortex. In this paper, we report spiking data recorded from the early visual cortex of a patient who was implanted with depth electrodes as part of her treatment for epilepsy. We were able to verify that human visual neurons have response properties similar to macaque neurons, including the size of their receptive fields and the presence of orientation tuning. We also found that the responses of human visual neurons are modulated by the visual context and by shifts of attention in a virtually identical manner to neurons in the macaque. This study, therefore, shows that the macaque visual system provides an excellent model for human visual cortical processing.

## Introduction

The early visual cortex consists of three areas, V1, V2, and V3, which provide a retinotopic map of the visual field. Our knowledge of the properties of neurons in early visual cortex derives largely from electrophysiological studies of animal models, including the cat, macaque monkey, and more recently, the mouse. The pioneering work of Hubel and Wiesel revealed that cells in early visual areas respond to visual stimuli in their receptive field, a circumscribed region of the retina. Visual cortical neurons are typically tuned for orientation [1] and spatial frequency [2] and give saturating responses when the contrast of the stimulus increases [3]. Later studies revealed that the neuronal responses in early visual cortex can also be modified by the context set by image elements outside the neurons’ receptive field. For example, texture-defined figures elicit stronger responses than textured backgrounds if the receptive field stimulus is held constant [4], and cognitive factors such as visual attention also influence the neuronal responses [5].

The usefulness of these data for our understanding of human vision depends on the similarities and differences between the animal models and the human [6]. So far, the comparison between animals and humans had to rely largely on post-mortem examinations to study the anatomy [7] and on indirect methods to measure brain activity such as functional magnetic resonance imaging (fMRI) [8], electroencephalography (EEG) [9], and magnetoencephalography (MEG) [10], with subdural electrocorticography (ECoG) as the most direct, yet invasive method [11]. Quantitative descriptions of the activity profiles of cells in early human visual cortex have been lacking. Early studies have reported visually driven spiking activity from visual cortex neurons (not localized to a particular area), but did not study them in great detail or quantify the receptive-field properties [12,13].

In this study, we report the properties of spiking activity recorded using microwires implanted in the occipital cortex of a patient during diagnostic surgery, part of her treatment for epilepsy. Most previous studies with microwires have targeted the medial temporal lobe of epileptic patients because this brain region is often implicated in the generation of epilepsy (e.g., [14–16]). Such recordings in visual cortex are much rarer as this region is almost never implicated in epileptogenesis. Here we report data from only two electrodes, and it is unlikely that we will be able to record more neurons from the same brain region in the near future. We measured the activity of neurons at these two electrodes in detail because the recordings were stable across a number of days. We could, therefore, for the first time to our knowledge, examine the tuning properties of the neurons in early visual cortex and explore how their activity is modulated by context and attention. We also recorded the local field potential (LFP) from the microwires, as recent data from patients implanted with ECoG grids suggest that the LFP can provide a first approximation of the tuning of spikes in visual cortex [11,17]. Our results demonstrate that spiking activity in human visual cortex shares many properties with that in macaque cortex, such as orientation and spatial frequency tuning, contrast saturation and surround suppression. We demonstrate, furthermore, that the spiking responses in early human visual cortex are enhanced by figure-ground segregation and object-based attention.

## Results

**Fig 1:**
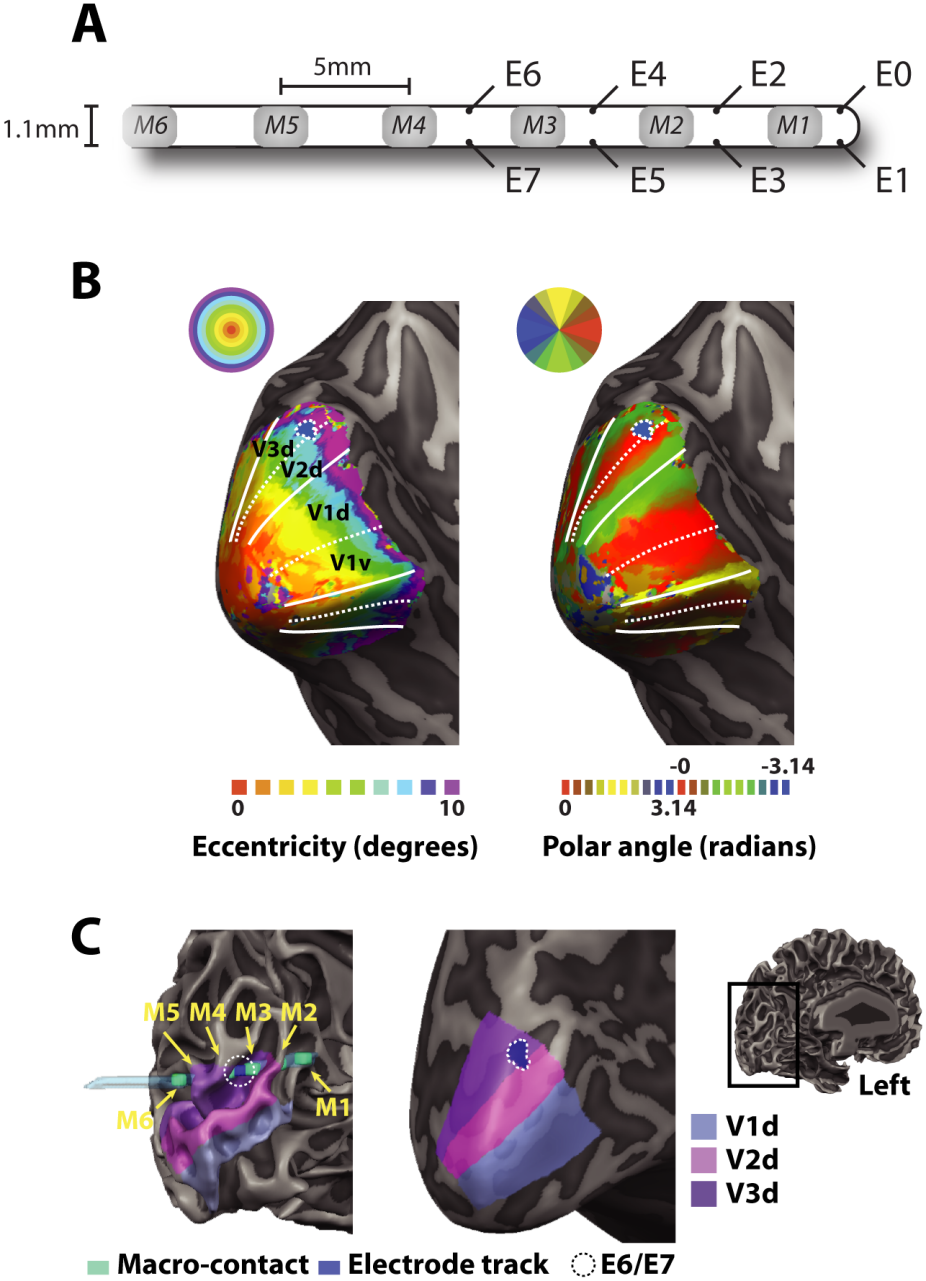
Localization of the microwires and retinotopy. (A) A schematic of the side-wire electrode. The body of the electrode was a hollow polyurethane tube. The gray-shaded regions depict the clinical macrocontacts used to measure intra-cranial EEG. The microwires were 40 μm diameter platinum-iridium wires situated between the macrocontacts. (B) Post-explantation fMRI measures of the retinotopic organization presented on an inflated representation of the patient’s left hemisphere. The left panel shows the eccentricity map and the right panel, the representation of polar angle. The solid lines indicate the representation of the vertical meridian and the dashed lines the representation of the horizontal meridian. The small blue patch outlined by a dashed line shows the estimated location of microwires E6 and E7 based on a spherical region-of-interest (diameter 5 mm) centered on the midpoint between macrocontacts M3 and M4 (see below). (C) (Left panel). The locations of the macrocontacts (turquoise regions) were obtained from a post-implantation CT scan, co-registered with a post-explantation structural MR image. The electrode track was clearly visible on the post-explantation scan as a region of signal dropout, depicted here in blue. The areas V1d–V3d are overlaid on the gray-white matter boundary. (Right panel) An inflated version of these areas and the estimated location of electrodes E6 and E7, which were situated very close to the representation of the horizontal meridian that marks the boundary between V2 and V3, and most likely within V3. Data is available from the Data Access Committee of the Netherlands Institute for Neuroscience: data-access@nin.knaw.nl.

We recorded the envelope of multi-unit spiking activity (MUA), thresholded spiking activity (MUAt), and LFPs from microwires situated in early visual cortex from the left hemisphere of a 35-y-old, female patient (Fig 1A and S1 Fig). The patient was implanted with clinical macroelectrodes in visual cortex (amongst other places) because she reported visual disturbances prior to the onset of her seizures. No visible lesions could be seen in pre-surgical MRI scans and the patient did not report further visual deficits before or after the depth electrode recordings. In the results, we will first describe the receptive field position and tuning of the neurons and then the effects of context and visual attention on neuronal activity.

### fMRI Localization of Electrode Sites

The early visual areas were mapped using standard retinotopic mapping techniques after explantation of the electrodes (Materials and Methods). We observed a normal retinotopic representation in both hemispheres (Fig 1B shows the left hemisphere). The positions of the microwires were judged from a post-implantation CT scan that was co-registered with a structural MRI image (Fig 1B and 1C). The CT scan was used to visualize the macrocontacts of the electrode. The microcontacts were not visible on the CT scan but were situated between the macrocontacts (Fig 1A). Electrodes E0–E3 were situated in or near the tip of the calcarine sulcus, most likely at very eccentric locations of V1, and electrodes E4 and E5 were situated in white matter. We obtained multi-unit recordings from electrodes E6 and E7, which were situated close to the representation of the horizontal meridian. Based on the location of the macroelectrodes, E6 and E7 were most likely located in V3 (Fig 1C), but we shall refer to their location as V2/V3 throughout this report because previous studies in monkeys reported that it is difficult to unambiguously assign electrodes sites to V2 or V3 if they are situated close to the representation of the horizontal meridian [18].

### Receptive Field Mapping and Response Latency

**Fig 2:**
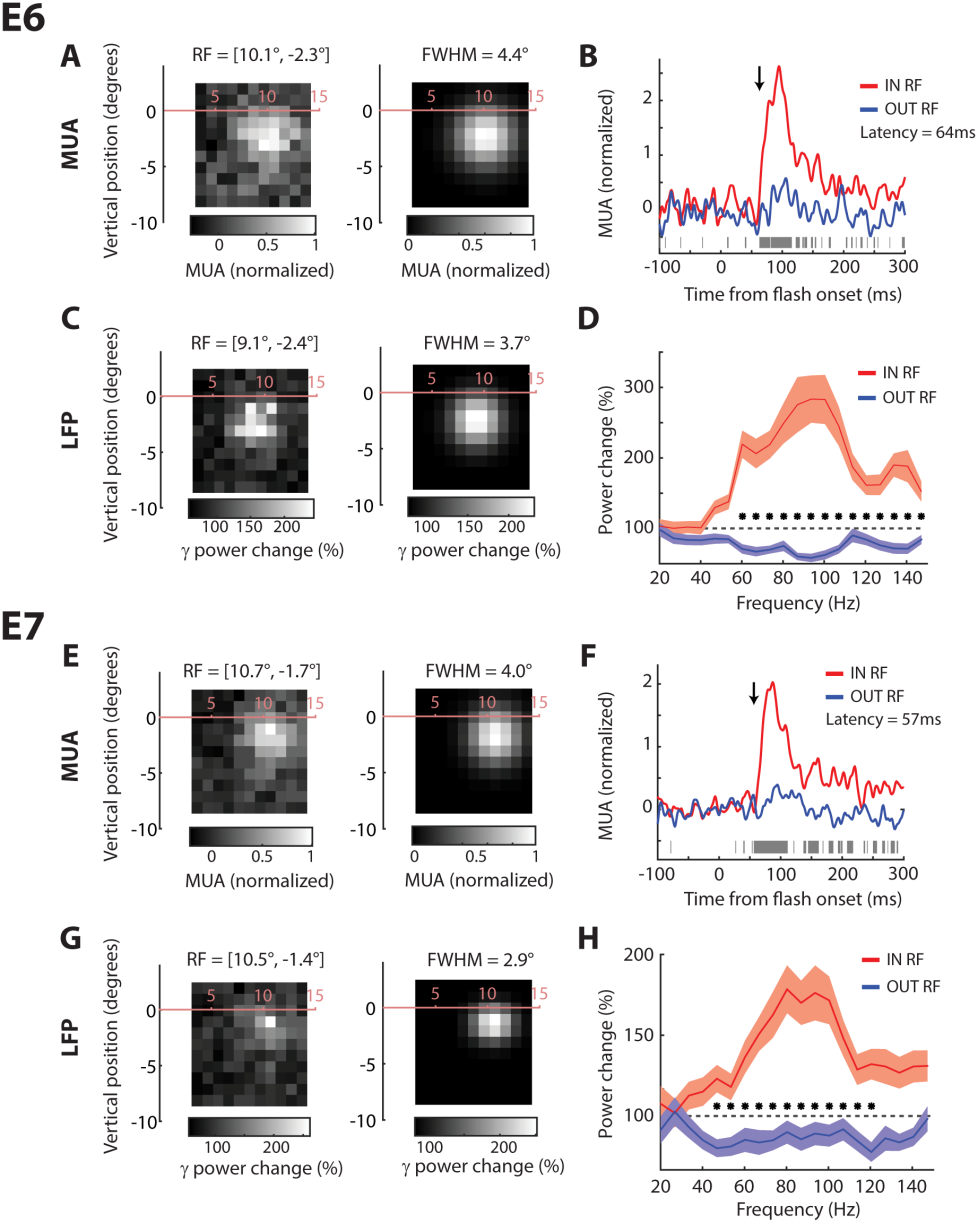
Receptive field location and size estimates from E6 and E7. (A) MUA receptive field (activity averaged between 50 and 300 ms after stimulus onset) from E6 to a 1° x 1° checkerboard briefly presented at each location of an 11 x 11 grid (left panel). The center of the RF was estimated by fitting a 2D Gaussian (right panel) to the data and the RF size as the width of the Gaussian at half of the maximum (FWHM), averaged across the *x* and *y* directions. (B) Responses from the 10% of locations closest to the center of the RF (red line) and the 10% of locations farthest from the center of the RF (blue line). The gray bars indicate samples with significant differences between these conditions (*t* test, *p* < 0.05). The latency of the response was estimated as the first significant sample that was followed by ten contiguous significant tests (arrow). (C) The change in gamma power (40–120 Hz) in a window from 100–250 ms after stimulus onset at each stimulus location relative to the average power across all locations (left panel). We also fit a 2D Gaussian to the spatial profile of the gamma power increase (right panel). (D) The relative increase in power when a flash was presented within the RF (10% closest locations, red line) compared to outside the RF (10% farthest locations, blue line). The shaded region depicts +/- 1 standard error of the mean (S.E.M) estimated by bootstrapping. (E–H) Data from electrode E7, same format as A–D. Data is available from doi:10.17605/OSF.IO/BRCZY

In our electrophysiological experiments we used an eye tracker and aborted trials whenever the patient’s gaze fell outside a 2° radius window centered on the fixation point. We first localized the multi-unit receptive fields (RFs) of electrodes E6 and E7 using a 1° × 1° checkerboard (check size: 0.33°) presented at each location of an 11° × 11° grid. The RFs were both located at an eccentricity of 10°–11°, close to the horizontal meridian (Fig 2A and 2E). We determined the average MUA responses at each location, in a 0.05–0.3 s window. We then fitted a 2D Gaussian and estimated the RF size as the full width at half maximum (FWHM) (Materials and Methods). Neurons at E6 had a RF-size of 4.4° and neurons at E7 had a slightly smaller RF of 4.0°. We observed similar RF properties when examining the thresholded multi-unit signal (S2A Fig). Previous studies have demonstrated that RF sizes of single-units in macaque V2/V3 at 10° eccentricity have an average value of 2°–3.5° (V2: refs [19,20]) or 3°–4.5° (V3: refs [19,21]) and multi-unit RFs in V2/V3 a size between 2°–3° (V2: ref [22]) and 4°–5° (V3: refs [23–25]). The RF sizes in human V2/V3 are therefore in good agreement with the results from macaque V3, increasing the likelihood that E6 and E7 were situated in V3.

A number of previous studies measured RFs in human visual cortex with the evoked potential or with the increase in power within the gamma band of intracranial ECoG recordings (e.g., [11,26]). To measure the influence of stimulus presentation on gamma power in the LFP, we computed the increase in power per frequency bin in a window from 100–250 ms after stimulus onset relative to the pre-stimulus epoch (150–0 ms before the stimulus). We observed that checks flashed in the RF caused a broad-band increase in gamma power (40–140 Hz) (Fig 2D and 2H). We used the increase in LFP gamma power to map the RFs. We observed clear RFs for E6 and E7 (Fig 2C and 2G), which overlapped with the MUA-RFs but were, to our surprise, slightly smaller (FWHM: E6, 3.7°; E7, 2.9°). Unlike previous reports [11], we could not measure clear RFs using the average event-related potential of the LFP (S3 Fig).

The latency of the multi-unit response elicited by luminance flashes in the RF was 64 ms for E6 and 57 ms for E7 (Fig 2B and 2F). These latencies are within the range of response latencies of single-units in macaque V2 and V3 [27] and also within the range of latencies of evoked potentials measured with ECoG grids [11].

### Tuning to Orientation, Direction, Spatial Frequency, and Contrast

Many neurons in the early visual cortices of mice, cats, and monkeys are tuned for the orientation, direction, spatial frequency, contrast, and size of visual stimuli. Studies using fMRI [8,28,29] have suggested that human visual cortex shares similar tuning characteristics, but spiking data is lacking. We therefore studied the tuning of neurons at electrodes E6 and E7 by presenting drifting sine-wave gratings (see Materials and Methods) in the RF while the patient maintained gaze at the fixation point.

**Fig 3:**
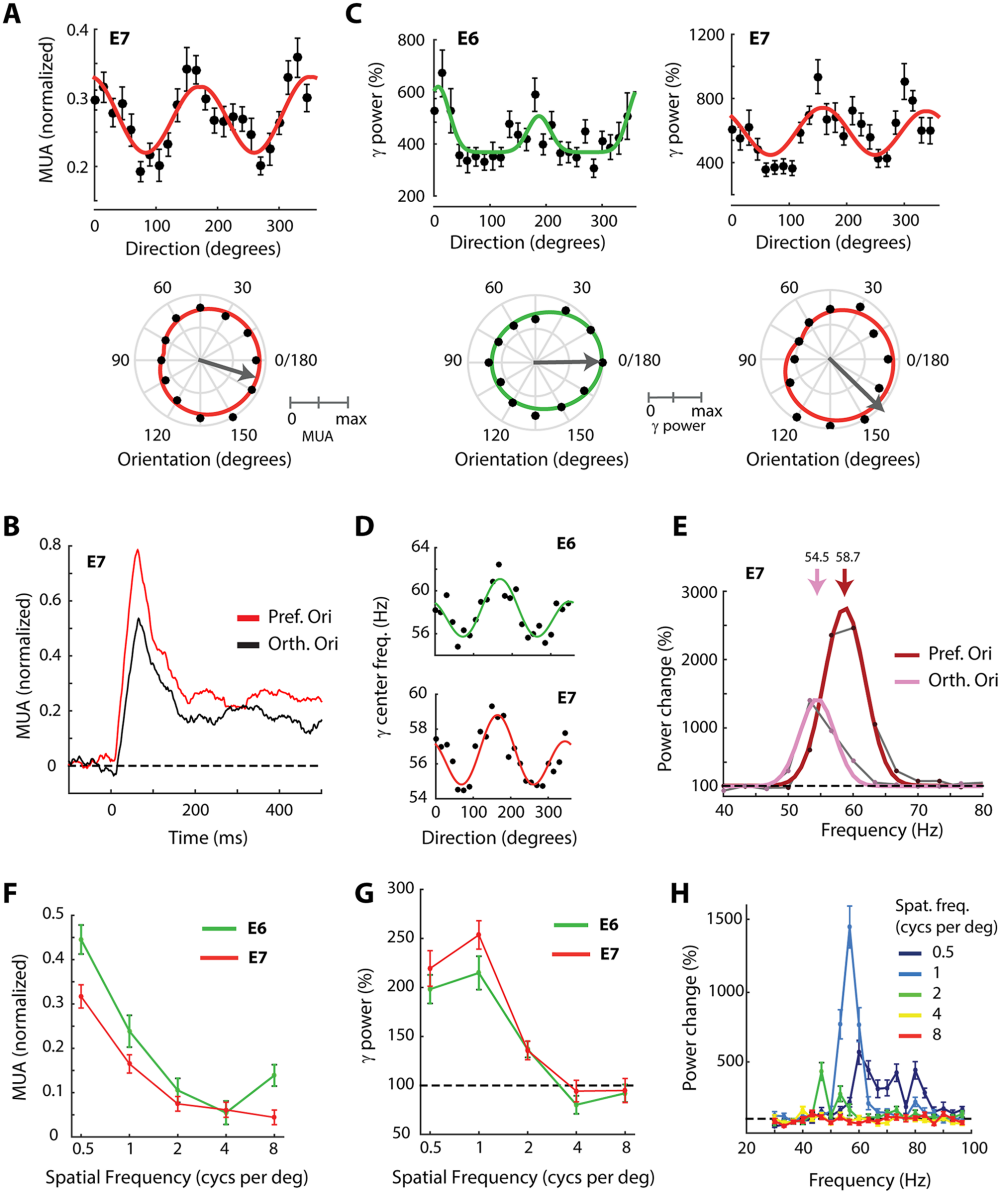
Orientation tuning in V2/V3. (A) Upper panel, MUA responses at electrode E7 to sine-wave gratings drifting in one of 24 directions. In this and subsequent figures, error-bars indicate +/- 1 S.E.M. The red line is the fit of a wrapped double Gaussian. The lower panel shows the same data in polar form, averaged across opposite movement directions with the same orientation and normalized to maximum response across orientations. The gray arrow is the preferred orientation (vector average). (B) Time course of MUA at E7 elicited by gratings of the preferred (averaged across 165° and 180°) and orthogonal (averaged across 75° and 90°) orientations. Orientation tuning arises very rapidly. (C) Upper panels: Gamma power (30–100 Hz) evoked by the 24 different directions at electrode E6 (green) and E7 (red) expressed as a percentage of the pre-stimulus baseline. The gamma power was significantly tuned for orientation but did not depend on direction. Lower panels: the same data in polar form, normalized to the maximum change in power across orientations. (D) The center frequency of the gamma peak at E6 and E7 at each of the 24 directions. (E) Change in power relative to pre-stimulus baseline in response to the preferred orientation (dark red) and the orthogonal orientation (pink) for E7. Note the presence of a clear peak in the power spectrum. The solid lines show a Gaussian fit, used to estimate the central frequency of the gamma peak. (F) Spatial frequency tuning of MUA at electrodes E6 (green) and E7 (red). (G) Gamma power (30–100 Hz) changes relative to the pre-stimulus baseline at different spatial frequencies. (H) Change in power relative to pre-stimulus baseline from E7 for each of the five spatial frequencies. Data is available from doi:10.17605/OSF.IO/BRCZY

We assessed orientation tuning strength using 1 –circular variance (1-CircVar) (Materials and Methods). This measure ranges from 0 (no tuning) to 1 (maximum tuning) and is more robust to noise than other measures such as the orientation tuning index [30]. The spiking activity of the neurons at electrode E7 was significantly tuned for orientation (Preferred orientation = 171.4°, i.e., close to vertical, 1-CircVar = 0.10, *p* < 0.001, Bootstrap test), but not for motion direction (1-CircVarDir = 0.02, *p* = 0.29, Bootstrap test) (Fig 3A and 3B). The thresholded multi-unit signal from E7 (MUAt) was also tuned for orientation (S2B Fig). The tuning width of the MUA signal was broad (half width at half maximum [HWHM] = 58°) but still within the range of single units in V2 and V3 of monkeys [31,32]. MUA from E6 was not significantly tuned to orientation (*p* = 0.38). We measured orientation tuning on two separate days, allowing us to assess the reliability of the signal across sessions (S4 Fig). We found that signals from E7 were significantly tuned for orientation in both sessions (Session 1: 1-CircVar = 0.10, *p* < 0.001, bootstrap test. Session 2: 1-CircVar = 0.09, *p* < 0.001, bootstrap test). The preferred orientation was very similar in both sessions (173° in session 1, 168° in session 2).

Previous studies demonstrated that the gamma power of the LFP in V1 of monkeys is also tuned to orientation [33,34]. The moving grating stimuli elicited a clear peak in the LFP power spectrum of human V2/V3, with a frequency between 50–60 Hz, and the power of this gamma oscillation at both electrodes was tuned for orientation (E6: 1-CircVar = 0.11, *p* < 0.001, HWHM = 28°. E7: 1-CircVar = 0.12, HWHM = 59°, *p* < 0.001) (Fig 3C). We also observed a reliable influence of stimulus orientation on the peak frequency of the gamma oscillation (determined by fitting a Gaussian function to the fractional increase in power per frequency bin; Fig 3D, Materials and Methods). Stimulus orientations eliciting strong gamma power caused oscillations with a higher frequency. Although the difference between the highest and lowest frequencies was only 4–5 Hz, the effect was very reliable (bootstrap test: *p* < 0.001 for both electrodes; Fig 3E). Thus, the power and frequency of the gamma oscillations were well tuned for orientation. The tuning of gamma matched the tuning of spikes at E7 because the preferred MUA orientation was 171°, the preferred orientation of gamma power was 158°, and gratings with an orientation of 167° evoked oscillations with the highest frequency.

Measurement of the spatial frequency tuning indicated that neurons at both E6 and E7 exhibited low-pass spatial frequency tuning (Fig 3F). The strongest responses were evoked by gratings of 0.5 cyc.degree^-1^, which was the lowest spatial frequency tested by us, so that we cannot exclude band-pass spatial frequency tuning, had the stimulus set contained even lower spatial frequencies. The spatial-frequency tuning of thresholded multi-unit activity (MUAt) was similar (S2C Fig). The gamma power of the LFP also varied with spatial frequency. Tuning curves obtained from gamma power in the 30–100 Hz range showed a moderate correspondence to those obtained with MUA with stronger power at low spatial frequencies (Fig 3G). The shape of the gamma peak in the power spectrum varied with spatial frequency (Fig 3H). The grating of 0.5 cyc.deg^-1^ produced a broad gamma peak at high frequencies (60–90 Hz) whereas higher spatial frequencies (1, 2 cyc.deg^-1^) produced tighter peaks at lower frequencies (40–60 Hz), and the highest spatial frequencies tested (4, 8 cyc.deg^-1^) did not produce any detectable gamma peak.

**Fig 4:**
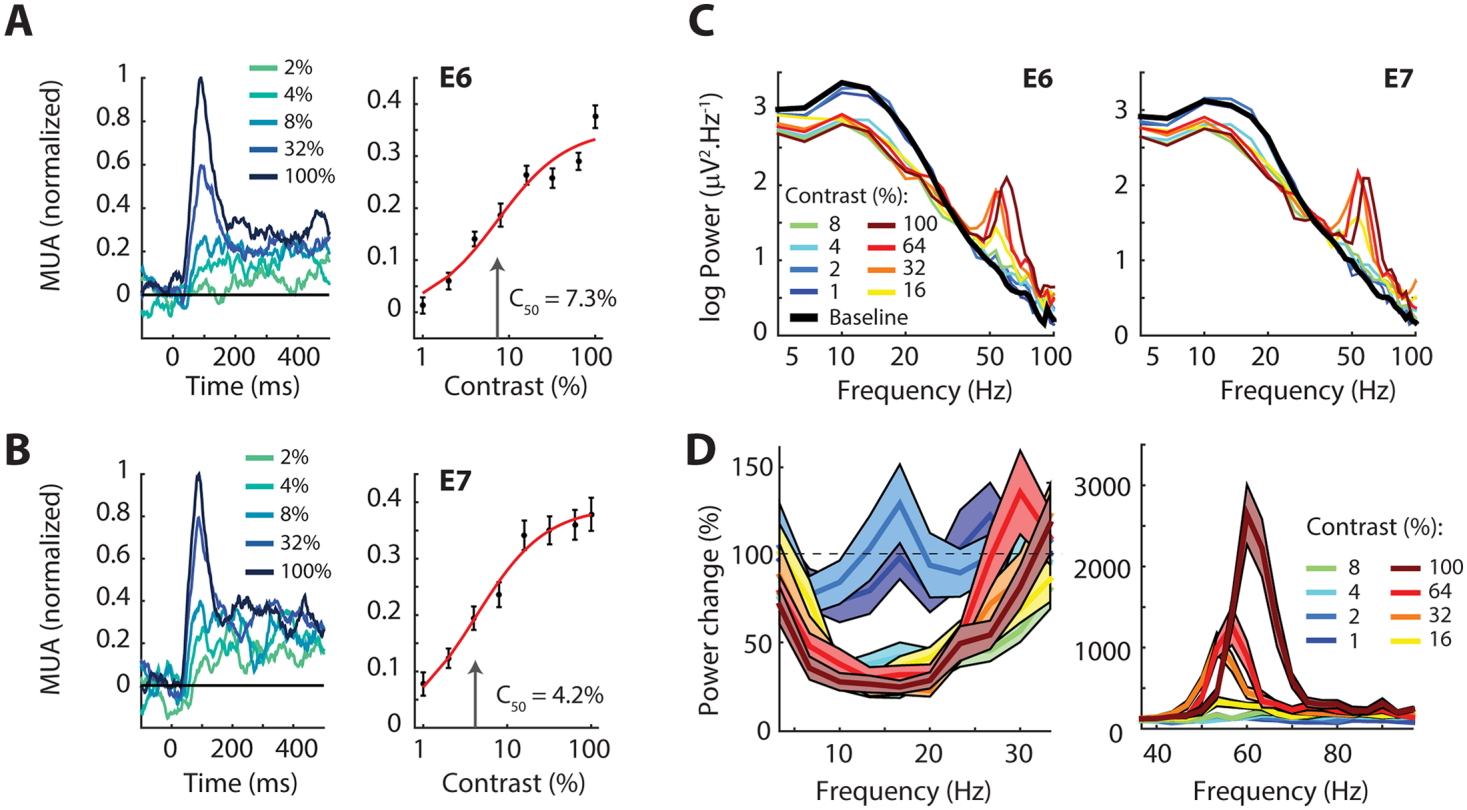
Contrast tuning of MUA and LFP. (A) Left panel, The MUA response evoked by drifting gratings of various contrasts at electrode E6. Gratings with a higher contrast evoked stronger MUA responses with a shorter latency. Right panel, the average MUA response in a window from 0–500 ms. The error-bars show +/- 1 S.E.M. The red line is the fit of the Naka-Rushton equation. The semi-saturation point (C_50_) is shown by the arrow. (B) Data from E7, the same format as in A. (C) Power spectra of the LFP of E6 and E7 elicited by gratings of different contrasts and the pre-stimulus baseline (black line). Contrasts above 2% suppress lower frequencies, and the highest contrasts induce a clear gamma peak. (D) The relative change in power for low frequencies (left panel) and high frequencies (right panel) compared to the pre-stimulus baseline, averaged across E6 and E7. Note the different scale on the *y*-axis in the two panels. The shaded regions are +/- 1 S.E.M. Data is available from doi:10.17605/OSF.IO/BRCZY

We next examined tuning to contrast with the drifting gratings. As expected, the MUA response increased with contrast and saturated at high contrasts (Fig 4A and 4B) (we obtained similar results with MUAt; S2D Fig). We fit a Naka-Rushton function to the contrast-response data to estimate the point at which MUA was 50% of its maximum (C_50_) and obtained a C_50_ of 7.3% for E6 and 4.2% for E7. These values are relatively low, yet within the range observed in the early visual cortex of monkeys [3,31,32]. The latency of the MUA response evoked by high-contrast gratings was shorter than that evoked by low-contrast gratings (Fig 4A and 4B), as has also been observed in monkey V1 [35,36]. We also examined the influence of contrast on the oscillations in the LFP. The amplitude of the gamma rhythm in the LFP of both E6 and E7 (Fig 4C) became stronger with increasing contrast (Fig 4C and 4D), and the peak frequency also became higher, in particular for the grating with 100% contrast, just as been observed in the visual cortex of monkeys [37–40]. At the same time, the grating stimuli with contrasts higher than 2% decreased LFP power at lower frequencies (10–25 Hz, the alpha to beta range) (Fig 4C and 4D), confirming results in monkey visual cortex [41,42].

### Size Tuning and Surround Suppression

**Fig 5:**
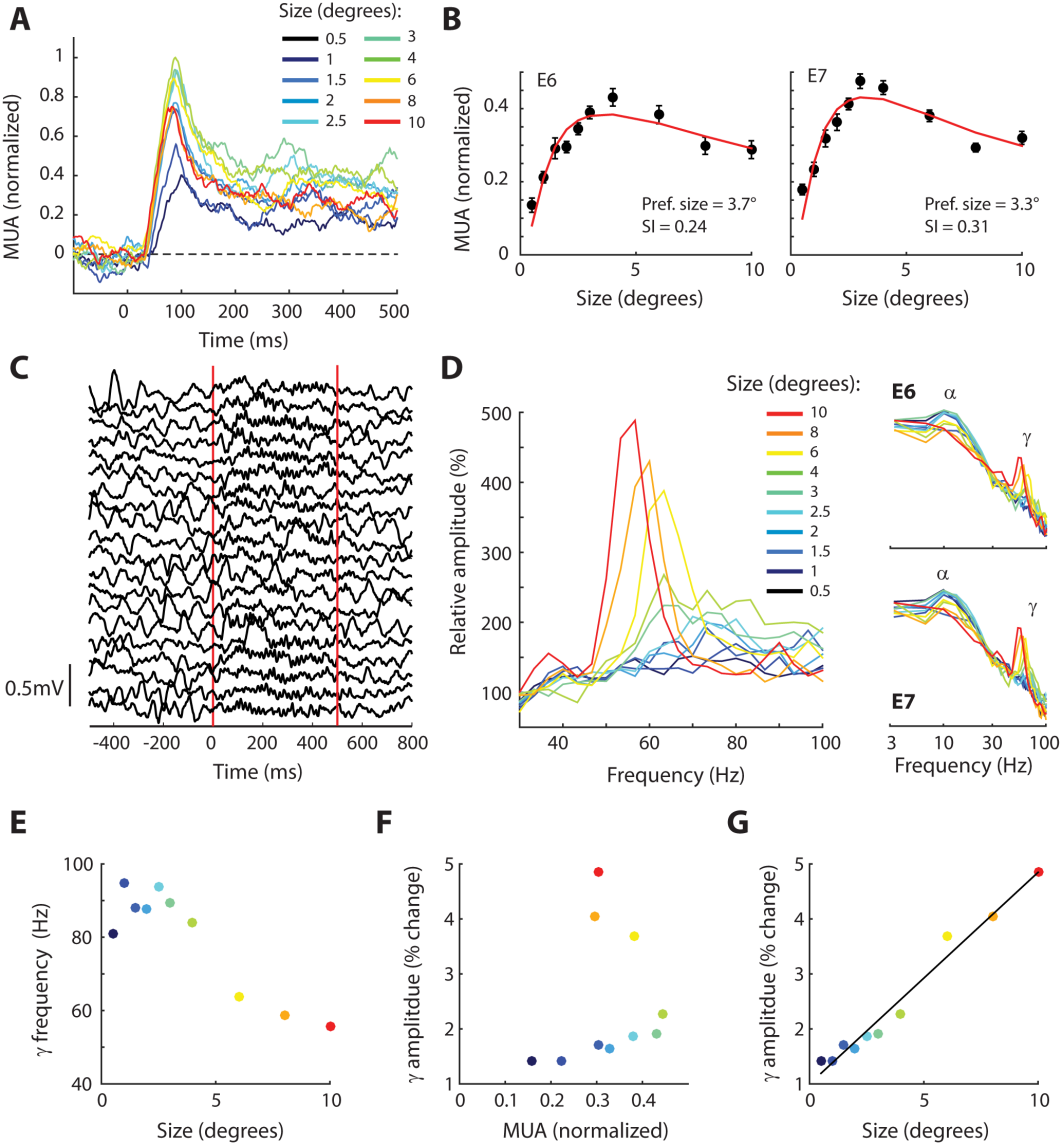
Size tuning of MUA and LFP. (A) MUA responses at E7 evoked by drifting gratings of various sizes. The peak response increases up to sizes around 3°–4° and activity is suppressed at larger sizes. (B) The average response between 0–500 ms at each size for E6 and E7. Error-bars show +/- 1 S.E.M. The red line is the best fit of the ratio-of-Gaussians model. (C) The traces show the raw LFP from 20 trials from E7. The red vertical lines mark the onset and offset of the stimulus (a 10° diameter grating). Note the low-frequency oscillations before the appearance of the stimulus, which are replaced with high-frequency gamma oscillations after stimulus onset. (D) The relative increase in the amplitude spectrum in the gamma range as function of stimulus size averaged across E6 and E7 (compared to a pre-stimulus baseline). The non-normalized (log) amplitude spectra for E6 and E7 are shown on the right. (E) Center frequency of the gamma peak, as estimated by fitting a Gaussian function. The colors of the dots indicate the size of the grating; conventions as in panel D. (F) Increase in gamma-band amplitude as a function of the MUA response. Note the absence of a clear relationship. (G) Dependence of the change in gamma amplitude on stimulus size. There was a strong correlation (r = 0.98, *p* < 0.001). Data is available from doi:10.17605/OSF.IO/BRCZY

Neurons in early visual cortex are tuned to the size of a visual stimulus. When the size of a stimulus is increased, neural responses are initially enhanced up to an optimum size. After this point, the response of many neurons decreases for larger sizes and it then reaches a stable level for very large sizes [43,44]. This response profile is well modeled by a ratio-of-Gaussians model with an excitatory receptive-field center and a larger, superimposed suppressive surround [44,45]. The responses of neurons at electrodes E6 and E7 were also tuned for the size of drifting gratings (Fig 5A) and their size-tuning profiles were well fit by the ratio-of-Gaussians model with optimum sizes of 3.7° (E6) and 3.3° (E7) (Fig 5B; the MUAt results were similar, see S2E Fig). These size-tuning profiles are in accordance with the size tuning of neurons in area V2 of the macaque monkey [46] and there is, to our knowledge, no data available from V3. We also computed the surround index (SI) to quantify the strength of the suppressive surround. We obtained SIs of 0.24 for E6 and 0.31 for E7, values which are also well within the range observed in V2 of monkeys [46].

We next examined the influence of stimulus size on the gamma oscillations of the LFP, because previous studies in monkeys demonstrated an influence of this factor on the strength and frequency of gamma oscillations [47,48]. The size of the grating stimulus had a strong influence on the LFP oscillations. The largest grating (10 degree diameter) suppressed the alpha oscillations that characterized the pre-stimulus epoch and replaced them with strong gamma oscillations, an effect visible in individual trials (Fig 5C). We fitted a Gaussian to the peak in the amplitude spectrum to examine the influence of stimulus size on the amplitude and frequency of the oscillation. Larger stimuli increased the amplitude of the gamma oscillations with a strong linear relationship between gamma amplitude and stimulus size (r^2^ = 0.96, *p* < 0.001) and they decreased the gamma frequency (Fig 5D, 5E and 5G). Thus, the influence of stimulus size on MUA (Fig 5B) differed from the effect on LFP gamma power (Fig 5D and 5G), and accordingly, the linear correlation between LFP gamma amplitude and the MUA response was not significant (r^2^ = 0.06, *p* = 0.49, Fig 5F). These results are in line with studies in area V1 of monkeys, which demonstrated that gamma power increases but spiking activity decreases when a grating stimulus encroaches into the suppressive surround of the receptive field [47,48].

### Contextual Modulation

The size tuning results illustrate that the responses of cells in the early visual cortex are modulated by contextual stimuli placed outside the RF [49], because surround stimuli suppress responses to a stimulus placed in the center of the RF. Previous studies revealed that surround suppression depends on the relative orientation of the center and surround; surround stimuli which share the same orientation as the center produce more suppression than stimuli with an orthogonal orientation [50–52]. This effect, known as orientation-tuned surround suppression (OTSS), is thought to enhance the representation of potential objects in the visual scene as it increases responses in regions with orientation contrast. Indeed, the perceived structure of the visual scene modulates neuronal activity in early visual cortex. Spiking activity elicited by figural regions is stronger than that elicited by background regions [4]. This figure-ground modulation (FGM) is delayed relative to the onset of the visual response and is thought to be due to feedback from higher visual areas [53,54].

**Fig 6:**
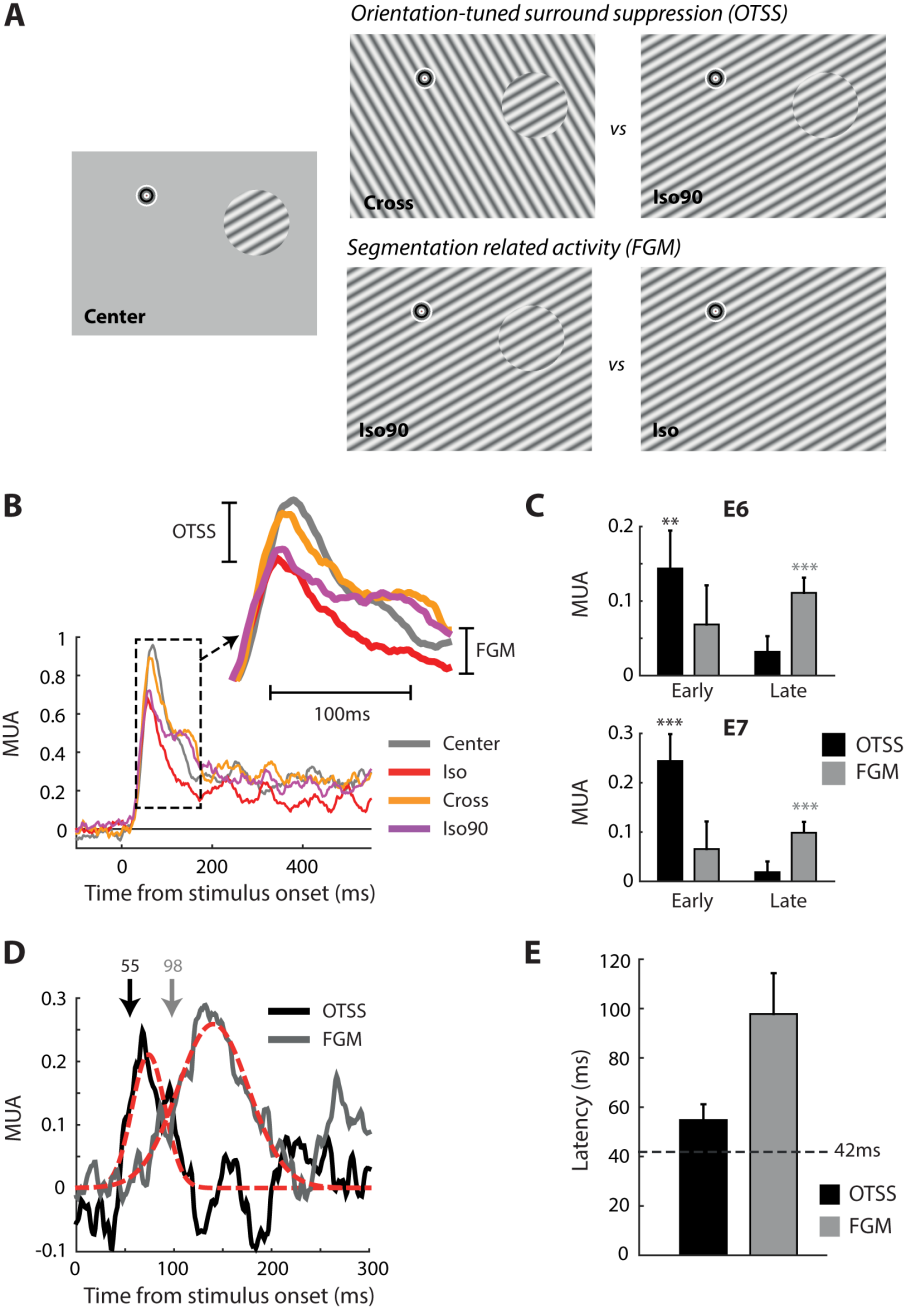
Contextual modulation of neuronal responses in human V2/V3. (A) The stimuli used to study contextual modulation. In the center-only condition, a 6° diameter grating was centered on the RF. We used two different orientations so that, on average, the orientation of the center was the same across conditions. In the Iso, Iso90, and Cross conditions, a full-screen surround was added. These conditions allowed extraction of signals related to two contextual effects: orientation-tuned surround suppression (upper panels) and figure-ground modulation (lower panels). (B) The MUA time courses averaged across E6 and E7. The inset shows responses during the early phase. These early responses were suppressed when the surround had the same orientation as the center (Iso, Iso90). Later activity depended on whether the central grating could be segmented from the background (Iso90, Cross) or not (Iso). (C) The average level of OTSS and FGM in an early (50–100 ms) and late (100–500 ms) time window for E6 and E7. Error bars represent 1 S.E.M. as estimated by a bootstrap procedure. Asterisks mark significant effects (** = *p* < 0.01, *** = *p* < 0.001, two-sample *t* test). (D) The time course of the modulatory effects. The black line illustrates OTSS (Cross-Iso90) averaged across E6 and E7. The gray line represents FGM (Iso90-Iso). The red lines show best-fitting Gaussian functions. Latencies were estimated as the point at which the Gaussians reached 50% of their maximum and are marked by the arrows. (E) Results of the latency analysis for OTSS and FGM. Error bars are 1 S.E.M. estimated by a bootstrap procedure. The black dashed line indicates the latency of the visual response estimated using the center-only condition. The latency was shorter than in Fig 2 because the effective contrast of the stimulus within the RF was higher. Data is available from doi:10.17605/OSF.IO/BRCZY

We studied contextual modulation in V2/V3 using a paradigm in which OTSS and FGM could be measured using the same set of stimuli. We presented stationary gratings with the same basic properties as those used in the previous section on size-tuning. Every stimulus contained a central grating of 6° diameter, which was either presented in isolation or surrounded by a full-screen grating (Fig 6A). The surround grating had the same orientation as the center (Iso, Iso90 conditions) or an orthogonal orientation (Cross condition). In the Iso90 condition, the phase of the surround grating was shifted by 90° to create a region that perceptually segregated from the background. To measure the influence of OTSS on neuronal activity, we compared the Cross to the Iso90 condition. Both stimuli induce a figure-ground percept but orientation contrast is only present in the Cross condition. To measure FGM we compared the Iso90 condition with the Iso condition. These two stimuli do not have orientation contrast, but figure-ground segmentation only occurs in the Iso90 condition.

The activity of neurons at E6 and E7 was strongly modulated by the surround gratings. The initial response was the same for all conditions, but after approximately 60 ms the response became suppressed in the Iso and Iso90 conditions relative to the Cross and Center only conditions (Fig 6B). Thus, in this early phase the suppression depended purely on the orientation contrast between center and surround. After approximately 100 ms, the response in the Iso90 condition became enhanced relative to the Iso condition, and it stayed at an elevated level throughout the remainder of the trial. In this late phase, the neural response therefore appeared to reflect the perceptual segregation of the center region from the background. For statistical analysis, we calculated OTSS and FGM in an early time window (50–100 ms) and a late time window (100–500 ms) (Fig 6C). In the early window, OTSS, calculated as the average difference between Cross and Iso90, was significantly greater than zero for both E6 and E7 (*t* tests, both ps < 0.01) but FGM was not (both ps > 0.1). In the late time window we observed the exact opposite pattern. Now FGM was significant (both ps < 0.001) whereas OTSS was not (both ps > 0.1). We estimated the latency of both effects by fitting a Gaussian function to the time course of the modulation (Fig 6D), averaging across the activity at E6 and E7 (which were very similar) (Fig 6E). The latency of OTSS, defined as the moment where the Gaussian function reached 50% of its maximum, was 55 ms, which was approximately 10 ms after the latency of the visual response in the center-only condition (dashed line in Fig 6E). FGM occurred at 98 ms, which was 43 ms after OTSS. These latencies are remarkably similar to those reported in monkey V1 for OTSS (e.g., ~60 ms in Bair et al. [55]) and FGM (~100 ms in Self et al., Lamme et al., and Poort et al. [54,56,57]). In our main experiments we used a 6° diameter center stimulus so that the boundary between the center and surround fell outside the RF, but we obtained virtually identical results with a smaller (4°) center stimulus that encroached into the RF (S5 Fig). Apparently, the presence of the boundary in the RF was not an important factor for OTSS and FGM.

These results, taken together, therefore suggest that the processes responsible for OTSS and FGM in the early visual cortex of humans are similar to those in monkeys.

### The Influence of Visual Attention

**Fig 7:**
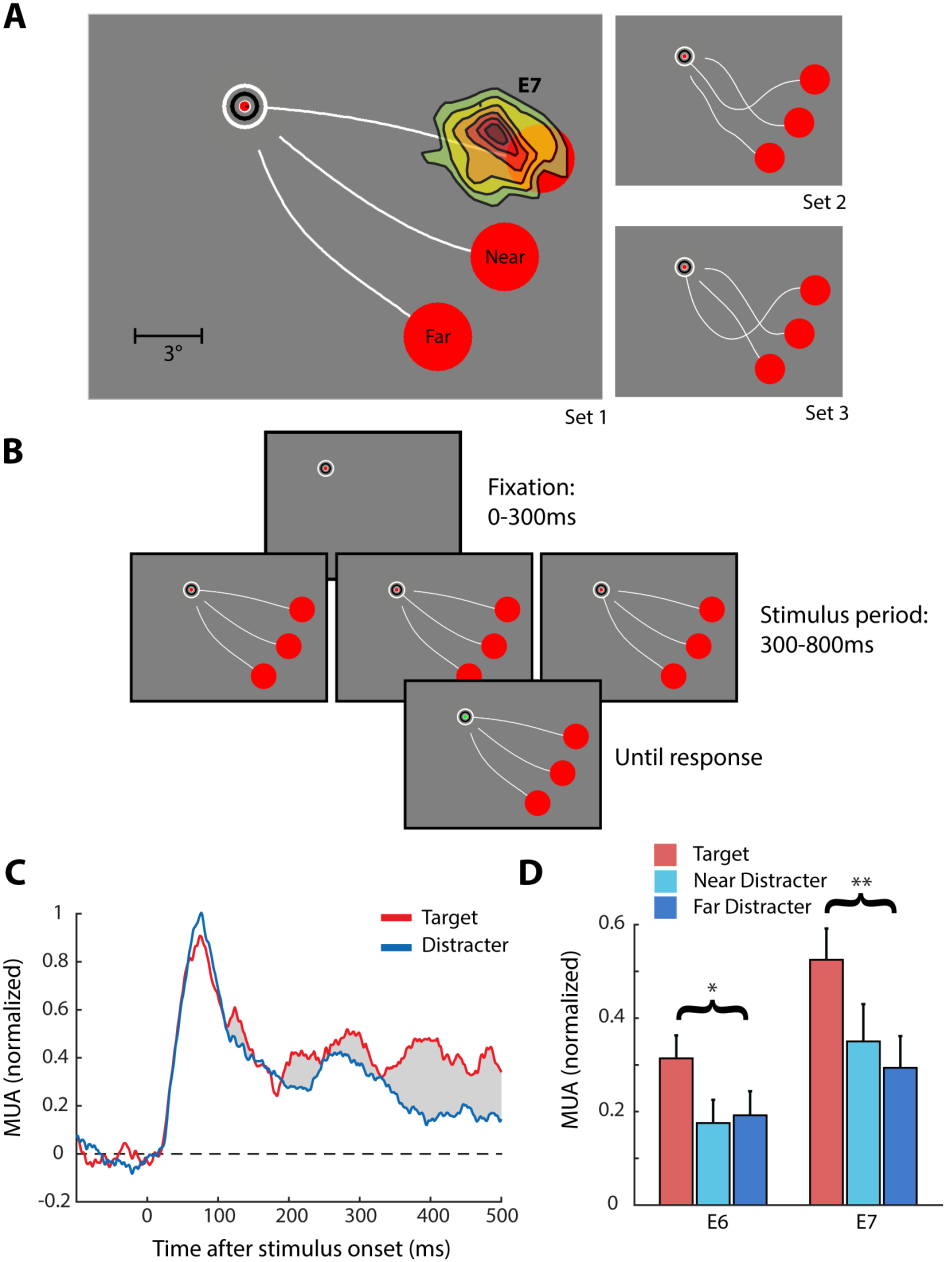
Attentional modulation of spiking activity in human V2/V3. (A) Example stimulus of the curve-tracing experiment. The RF of MUA at E7 is also shown. There were two distractor curves, which were connected to two other circles near and far from the RF. The right panels illustrate the two alternative stimulus configurations. (B) The stimulus paradigm: The patient initially had to fixate for 300 ms. One of nine possible stimulus configurations was shown, the three curve configurations of stimulus set 1 are shown here. The patient had to mentally trace the target curve attached to the fixation point. After a delay of 500 ms the fixation point turned green and the patient was required to make a saccade towards the red target circle connected to the fixation dot. (C) The MUA response averaged across E6 and E7 when the RFs fell on the target curve (red line) or on one of the two distractor curves (blue line, averaged across near and far distractor). The attentional modulation is the difference between the two responses (gray shading). (D) The average response at E6 and E7 in a window from 200 to 500 ms elicited by the target curve (red bar) and the distractors (blue bars). The asterisks indicate the significance of the difference in MUA between the target and distractor conditions as assessed by a *t* test (* = *p* < 0.05, ** = *p* < 0.01). Error-bars indicate 1 S.E.M. Data is available from doi:10.17605/OSF.IO/BRCZY

Previous studies demonstrated that the activity of neurons in early visual cortex of monkeys does not only depend on the stimulus in the receptive field and the surround but also on behavioral relevance. Specifically, attention shifts towards a stimulus in the receptive field enhance neuronal activity [5,58,59]. fMRI, EEG, and ECoG studies in humans support these findings [60–62], but some of them reported that the influence of attention is weak [17] and these methods could not resolve whether the attentional effect causes subthreshold membrane potential changes or if it also leads to changes in spiking activity. To test the influence of attention on the spiking activity in human V2/V3 we used a curve-tracing task (Fig 7A), in which the patient mentally traced a target curve that started at the fixation point to locate a larger red circle at the other end of this curve. Previous psychophysical studies demonstrated that human subjects gradually spread object-based attention over such a target curve [63]. Studies in monkeys revealed a neuronal correlate of the spread of object-based attention because traced curves elicit stronger spiking activity in early visual cortex than curves that are ignored [5,64].

When the stimulus appeared on the screen, the patient traced the target curve connected to the fixation point while maintaining gaze within a 2° radius window centered on the fixation point. After a delay of 500 ms, the fixation point changed color from red to green, and this was the cue to make an eye movement to a window centered on the larger circle at the end of this curve (Fig 7A). The total number of stimuli was nine, because each of the three curves could be connected to the fixation point with equal probability, and we presented a total of three configurations that varied in the number of crossings between the curves. We first gave the patient a few minutes to practice before we started the recordings. After these practice trials, the patient performed the task with an accuracy of 90%; on 4% of trials she selected the wrong eye-movement target, and on the remaining 6% of trials the saccade did not enter any of the eye movement windows. We examined spiking activity of neurons at E6 and E7 on correct trials during the fixation period when the patient maintained gaze on the fixation point (Fig 7B) and excluded trials in which the patient made microsaccades during this period (maximum velocity threshold of 10°.s^-1^ maintained for at least 10 ms; see S6 Fig for details of the eye movement analysis). Because the total number of trials was relatively small (*n* = 135 correct trials) we averaged the neuronal responses on correct trials across the three stimulus configurations. The initial, visually driven response did not distinguish between the target and distractor curves (time window, 0–150 ms, *t* test, E6: *p* = 0.41, E7: *p* = 0.32). However, in a later response period (200–500 ms), the MUA responses evoked by the target curve were significantly stronger than those evoked by the distractor curves (*t* test, E6: *p* = 0.04, E7: *p* = 0.01). The activity elicited by the distractor curve did not depend on whether the target curve was adjacent to the receptive field or farther away (*t* test, E6: *p* = 0.84, E7: *p* = 0.61, Fig 7D). We also examined whether small changes in eye position or speed within the fixation window could account for the difference in activity evoked by the target and distractor curves, but the mean eye position and eye velocity were similar (U-tests: *x*-position, *p* = 0.33; *y*-position, *p* = 0.16; velocity, *p* = 0.33) (S6 Fig). Thus, visual attention modulates spiking activity in human area V2/V3.

## Discussion

To our knowledge, the results described above represent the first quantitative analysis of the spiking responses of cells in the early visual cortex of humans. We found an excellent correspondence between the tuning of neurons in human V2/V3 and the tuning of neurons in monkey early visual cortex at similar eccentricities. Perhaps even more strikingly, we observed that spiking responses were modulated by contextual stimuli and attention with similar amplitudes and latencies to those observed in the early visual cortex of the macaque. We also observed several phenomena in the gamma band of the LFP, which further confirmed the similarity in neuronal activity between macaques and humans.

### Comparison of the Selectivity of Spiking Activity in Humans and Macaque

One of our aims was to compare the spiking activity in human and macaque early visual cortex. In making this comparison, we were faced with the difficulty that most recordings in macaque early visual cortex have been performed in V1, some in V2, and only relatively few in V3. Furthermore, the RFs measured by us had an eccentricity of 10°, where recordings in macaques are rarely made. The many correspondences are nevertheless noteworthy. The size of the RFs were similar to those reported for MUA in V3 [23–25]. Contrast semi-saturation values (C_50_) were 4%–8%, which is within the range expected in V1 [3], V2 [31], and V3 [32]. Furthermore, the strength of surround suppression was compatible with that in macaque V2 and the preferred stimulus size was similar [46]. Taken together, these results suggest that the tuning of cells in the early visual cortex of humans is well approximated by that in macaques.

### Gamma Oscillations

We observed large visually driven increases in gamma-band power of the LFP that were visible in individual trials (Fig 5C). Brief flashes that we used to map the RFs elicited broad-band gamma oscillations from a region that had a similar size as the MUA RF. Thus, the gamma band of the LFP can be used to estimate the RF location and size if spiking data is not available. Moving grating stimuli elicited gamma oscillations in the LFP with power that was restricted to a narrower frequency band than the flashing stimulus [65]. The power and frequency of these narrow-band oscillations depended on the orientation, contrast, and size of the grating. The optimal orientation elicited oscillations that had more power and a higher frequency than stimuli with suboptimal orientations, in accordance with studies in cat [66] and macaque V1 [33,48,67]. High-contrast stimuli produced large increases in gamma power [37], and the very highest contrasts led to oscillations at higher frequencies, as has been reported in macaque V1 [37,39,40] and V2 [40].

When we varied the orientation or contrast of the stimulus, increases in MUA were associated with increases in gamma frequency and power. However, the tuning of MUA and gamma to stimulus size differed. Larger grating stimuli suppressed the MUA and caused gamma oscillations with a lower frequency, but the gamma power increased with stimulus size, in accordance with results from macaque V1 [46]. The underlying mechanisms leading to this dissociation remain unknown, but it has been suggested that the strength of gamma oscillations depends critically on the balance between excitation and inhibition in cortex [47,48,68–70]. Thus, the tuning of the gamma-band oscillations in humans closely resembles that in the macaque. This generalization from the macaque to the human is important because insights into the relationship between gamma and spiking in macaques may facilitate the interpretation of ECoG, MEG, and EEG data in humans and its relation with the tuning of cells. It should be noted, however, that grating stimuli evoke strong gamma oscillations, whereas complex, more naturalistic stimuli evoke weaker gamma oscillations. Thus, the relationship between gamma and the firing of neurons is stimulus dependent [65].

### Modulation of Spiking by Visual Context and Attention

We observed that responses in human V2/V3 exhibit two well-known contextual effects: orientation-tuned surround-suppression and figure-ground modulation (Fig 6). Image regions with orientation contrast and regions that belonged to figures elicited stronger spiking responses than image regions with a uniform orientation, even if the stimulus in the RF was held constant. The time courses of these contextual effects were very similar to those reported in macaque V1 [55,56]. This result suggests that V2/V3 in humans is targeted by feedback from higher visual areas that carry contextual information from larger regions of the visual scene, just like V1 in the monkey [54,71–73]. Our results demonstrate that cells in early visual cortex in humans do not only extract local feature information but that they may also play an active role in perceptual processes such as scene segmentation and figure-ground assignment, and they thereby confirm previous EEG and fMRI studies [74,75].

The attentional modulation that we observed in a curve-tracing task (Fig 7) presents another striking similarity with previous results in the visual cortex of monkeys. Just as in monkey V1 [5], the initial response of the neurons in human V2/V3 did not discriminate between a target and a distractor curve, but after a delay, the response elicited by the target curve was enhanced. Psychophysical studies [63,76] demonstrated that human observers spread object-based attention across the target curve and the modulation of neuronal responses in V2/V3 can therefore be explained by the spread of attention [77].

### Limitations

One possible concern in the interpretation of these results is that the data came from an epileptic patient who reported visual disturbances prior to the onset of the seizure. However it is unlikely that the cortical region we were recording from was structurally abnormal, for a number of reasons. Firstly, although the patient reported that a feeling of visual movement or “fluttering” sometimes preceded the seizures, they were not triggered by visual stimuli, and her visual performance and acuity were normal. Secondly, we observed no lesions or abnormalities in the anatomical MRI images or functional maps of her visual cortex. Thirdly, the clinical analysis of the intracranial EEG recordings suggested an onset zone outside of visual cortex close to the temporo-parietal junction. Lastly, the tuning of the neurons to visual stimuli corresponded well to those observed in the monkey, which seems unlikely for grossly abnormal or damaged cortical circuitry.

We recorded from only two microwires in V2/V3, and we do not foresee opportunities to increase the size of our sample in the near future, given the extreme rarity with which depth electrodes are placed in early visual cortex. At the same time, we were able to carry out many tests for the neurons at these two recording electrodes, because the recordings were stable across a number of days. Another limitation is that we recorded MUA rather than the activity of single neurons. The tuning of MUA reflects the average across a population of neurons in the vicinity of the electrodes in V2/V3. On the one hand, tuning to stimulus direction and orientation was presumably less sharp than that of single neurons, because MUA implicitly averages activity across a number of neurons with different tuning curves. This limitation is less severe for tuning to size or contrast, because pooling across neurons does provide insight into the average dependence of firing rates within a cortical region on these factors. This also holds true for contextual and attentional effects. Contextual effects in early visual cortex are highly consistent across neurons [4], and attention usually increases neuronal firing rates so that averaging across cells provides insight into the magnitude of the average effect [78].

### Conclusions

The data presented here represents the first quantitative study of spiking activity in human early visual cortex. Using several different visual paradigms, we found remarkable similarities between the spiking of human visual neurons and those in macaque visual cortex, both in the basic tuning properties as well as in contextual modulation and attentional paradigms. These results confirm that the macaque monkey is an excellent model system for studying the properties of cells in the early visual system of humans.

## Materials and Methods

### Surgical Procedures and Patient Details

The study was approved by the medical ethics board of the Free University Medical Center (protocol 2009/194). The patient gave written informed consent to participate in the study. She was a 35-y-old female with a normal IQ and no reported visual deficits. The patient had late-onset epilepsy without any relevant antecedents. Her semeiology (seizure symptoms) strongly suggested the involvement of posterior temporal cortex, or the visual cortex without clear lateralization. She was implanted with 15 depth electrodes (AdTech) under general anesthesia using frameless stereotaxy. The electrodes were inserted through a guide-tube under the guidance of an online stereotactic positioning system. Five electrodes were placed in the right hemisphere, and nine electrodes in the left hemisphere (to determine lateralization of the seizure onset). One electrode in visual cortex was selected to contain a microwire bundle. Recordings from the microwires began 2 d after the surgery and proceeded for a further 7 d.

### Electrophysiological Procedures

The impedance of the side-wire electrodes was measured post-explantation as 158 KΩ (E6) and 115 KΩ (E7). Signals from the microwires were amplified with respect to a skull-screw ground using a unity gain HS-9 head-stage amplifier (NeuraLynx). We digitized and sampled the signal at 32.5 kHz before storing it for later analysis. The raw signals from E6 and E7 were re-referenced to the average of the (nonspiking) electrodes E0–E4 (E5 was excluded from the average due to high noise levels). From the re-referenced signal we created three signals: the local field potential (LFP), the envelope of multi-unit activity (MUA) and the thresholded multi-unit activity (MUAt). The LFP was created by first down-sampling to 930 Hz, then band-pass filtering the resulting signal between 1 Hz and 200 Hz using a second order, zero-phase Butterworth filter. Line-noise was removed by fitting a 50 Hz sine-wave to each individual trial, then subtracting it. S1 Fig outlines our procedure for generating MUA and MUAt. Briefly, we measured MUA by band-passing the raw signal between 500 Hz and 5 kHz to isolate high-frequency (spiking) activity. This filtered signal was rectified (negative becomes positive), down-sampled to 930 Hz and low-pass filtered (<200 Hz) to measure the envelope of the spiking activity. The MUA provides an instantaneous, threshold-free estimate of spiking activity in the vicinity of the microwire, and it is a good measure of the average single-cell activity [79] within 150 μm of the electrode tip [54]. We also generated MUAt by thresholding the band-passed signal (see S1 Fig for details).

### Visual Stimuli

We generated visual stimuli in MATLAB using the COGENT graphics toolbox developed by John Romaya at the LON at the Wellcome Department of Imaging Neuroscience and custom scripts. Stimuli were presented at 60 Hz on a laptop LCD screen (26 cm width) located at a distance of 66 cm from the patient. The LCD screen had a mean luminance of 40 cd.m^-2^ and this value was used as the background gray in all experiments except where indicated. The patient viewed the screen in a dimly lit room with her head on a chin rest, and we measured her eye movements with an Eyelink T1000 system sampling at 1,000 Hz. In all sessions, the patient began each trial by fixating on a circular fixation pattern presented towards the top-left corner of the laptop screen (so that we could place stimuli in the neurons’ RFs). She maintained fixation within a 2° radius circular window during presentation of the stimuli; otherwise, the trial was aborted. Analysis of her eye position showed that she actually maintained fixation in a much smaller area than this (S6 Fig). For the experiments measuring contextual and attentional modulation, we discarded trials in which the eye position was further than 1° from the center of fixation (10%–15% of trials).

We measured RFs by flashing a small black-and-white checkerboard pattern (1° x 1° size, check-size 0.33°) at every point of an 11° x 11° grid. RF tuning properties were measured using drifting sine-wave gratings placed at a location that activated neurons at both E6 and E7 (10.3° eccentricity, -14° angle from horizontal meridian). As standard stimuli, we used gratings with a Michelson contrast of 80% that were 10° in diameter with a spatial frequency of 1 cycle/degree and drifted with a temporal frequency of 3 Hz. We presented orientations of 45° or 135°, except in the orientation/direction tuning session, which included 24 directions ranging from 0 to 345°, in steps of 15°. For the other tuning sessions, we varied one parameter while holding the others constant. Each session contained ten repeats of every stimulus parameter. We completed two sessions for the orientation-tuning and contrast-tuning experiments and one session for the spatial frequency and size tuning experiments.

In the contextual modulation experiments, the gratings had the same properties as those described above except that they were stationary. The phase of the central grating was randomly chosen on each trial from a uniform distribution ranging from -π to π. We used two different, orthogonal orientations for the central grating, 60° and 150°. We presented an equal number of trials with the two orientations so that the average stimulation of the RF was identical for all conditions. The stimulus duration was 500 ms with an inter-trial interval of 500 ms.

In the curve-tracing experiments the patient began each trial by directing gaze to the fixation point. After 300 ms the curves and targets appeared. The curves had a width of 5 pixels (0.1°). The targets were red, 3° in diameter and were presented at -10°, -30°, and -50° from the horizontal meridian at 13° eccentricity. We presented a total of nine stimuli in a pseudo-random sequence (Fig 7A). The patient had to maintain fixation for a further 500 ms, and then the fixation point became green, cueing the patient to make a saccade to the larger circle at the end of the target curve.

### Data Analysis

For the MUA analysis, we first subtracted the average background activity across all trials (-0.2 s to 0 s before stimulus onset). The data from the RF mapping experiment (shown in Fig 2) were normalized by dividing by the average activity in a window from 0.05–0.3 s from the position that gave the strongest response. All other MUA data were normalized by dividing by the peak response in a time window between 0.03 and 0.15 s. In the RF tuning experiments we normalized to the maximum activity across conditions, in the contextual modulation experiment to the maximum response in the center-only condition, and in the curve-tracing experiments to the maximum response averaged across all trials. For MUAt analysis we first created histograms by binning the spike-times from each individual trial into bins of 1.1 ms duration. We then convolved the resulting spike-trains with a Gaussian density function to create spike-density functions. See S1 Fig for further details. For LFP analysis of the RF tuning data, we computed the Fourier transform of the LFP in two windows, a pre-trial baseline (-0.3 to 0 s before stimulus onset) and during the stationary period of the response (0.15–0.45 s after stimulus onset). We applied a Hann window before computing the Fourier transform to reduce edge artifacts. We estimated the power in each frequency bin as the squared magnitude of the signal and computed the relative change in power by dividing the post-stimulus power by the baseline power spectrum.

### MRI Localization of the Electrode Contacts

The patient was scanned three months after the explantation of the electrodes on a 3T MRI scanner (Prisma Fit; Siemens Medical Systems, Erlangen, Germany) equipped with a 64-channel head coil.

We collected anatomical T1-weighted images using a magnetization-prepared rapid-acquisition gradient echo (MPRAGE) pulse sequence (192 sagittal slices; Repetition Time [TR] = 2,250 ms; Echo Time [TE] = 2.21 ms; Flip Angle [FA] = 9°; Field of View [FoV] = 256 × 256 mm^2^; 1 mm isotropic resolution; GRAPPA = 2). Functional data were acquired using a gradient-echo echo-planar imaging sequence (30 transversal slices; TR = 1,000 ms; TE = 30 ms; FA = 60°; FoV = 216 × 216 mm^2^; 2 mm isotropic resolution; no slice gap; MultiBand factor = 2; GRAPPA = 2).

We analyzed the imaging data with BrainVoyager QX (v2.8.4; Brain Innovation, Maastricht, the Netherlands). Anatomical data underwent brain extraction, followed by inhomogeneity correction and semi-automatic segmentation of the gray-white matter boundary for mesh reconstruction of the cortical surface. Preprocessing of the functional data included slice scan time correction, (rigid body) motion correction, and temporal high-pass filtering (up to two cycles per run). To estimate the location of the electrode contacts, the post-implantation CT scan was aligned to the anatomical MR data, and the clearly visible macrocontacts were segmented for visualization in combination with the subject’s cortex mesh reconstruction. Functional localization relied on delineation of early visual areas based on three population Receptive Field (pRF) mapping runs (each lasting ~4 min) [80]. In every run, a bar stimulus (projected onto a back-projection screen visible to the subject via a mirror mounted onto the head coil) was semi-randomly presented twice for 2 s at 12 different locations and four orientations (spanning a total of 20° by 20°) using the StimulGL presentation software [81].

### Fitting Receptive Field Data

For the analysis of the receptive-field location, we took the average activity (either MUA or gamma power) in response to each flash location in a time window between 50–300 ms (MUA) or 100–250 ms (gamma power). The responses, *R*(*x*,*y*), at each *x* and *y* location were normalized by dividing by the maximum response across all locations, and we fit a 2D, elliptical Gaussian using nonlinear multidimensional minimization:

$$R(x,y)\;=\;e^{-a{\left(x-{rf}_{x}\right)}^{2}+2b(x-{rf}_{x})(y-{rf}_{y})+c{\left(y-{rf}_{y}\right)}^{2}}$$

$$where\;a\;=\;\frac{{cos}^{2}\theta }{2\sigma_{x}^{2}}+\frac{{sin}^{2}\theta }{2\sigma_{y}^{2}},\;b\;=\;-\frac{sin2\theta }{4\sigma_{x}^{2}}+\frac{sin2\theta }{4\sigma_{y}^{2}}\;and\;c\;=\;\frac{{sin}^{2}\theta }{2\sigma_{x}^{2}}+\frac{{cos}^{2}\theta }{2\sigma_{y}^{2}}.$$

The center of the Gaussian (*rf*_*x*_,*rf*_*y*_), the standard deviations in the *x* and *y* directions, *σ*_*x*_ and *σ*_*y*_ and the orientation of the Gaussian *θ* are the free parameters.

### Fitting RF Tuning Properties

Orientation tuning width was assessed by fitting a circular Gaussian using nonlinear least-squares fitting in MATLAB as follows:

$$R(\theta )\;=\;C+\;R_{p}∙exp(-\frac{{{ang}_{ori}(\theta -\theta_{pref})}^{2}}{{2\sigma }^{2}}),$$

Where *R*(*θ*) is the response between 0–0.5 s for orientation *θ*, *C* is the offset, *R*_*p*_ is the response to the preferred orientation, *ang*_*ori*_(*x*) = min(abs(x),abs(x-180),abs(x+180)) wraps angular differences to the interval 0° to 90°, and *σ* is the standard deviation of the Gaussian. Tuning width was measured as the orientation difference between the peak of the response and the midpoint between the peak and the minimum value (half width at half maximum, HWHM). Tuning strength was assessed using *1-CircVar* [82] which has been shown to be a more robust estimator of tuning strength than orientation indices [30]. It was calculated as follows:

$$1-CircVar\;=\;\left|\frac{\sum_{k}R(\theta_{k})e^{\left(2i\theta_{k}\right)}}{\sum_{k}R(\theta_{k})}\right|$$

where *R*(*θ*_*k*_) is the response to the orientation *θ*_*k*_ (in radians). The significance of the tuning strength was assessed by bootstrapping. For each electrode, we randomly resampled with replacement an equal number of trials as in the original dataset but shuffled the responses across orientations and calculated 1-CircVar 10,000 times to create the expected null distribution of the 1-CircVar statistic from which we derived the *p*-value.

We fitted a Naka-Rushton equation to the contrast response curves in a time window from 0 to 0.5 s:

$$R(x)\;=\;\frac{{bx}^{N}}{{C_{50}}^{N}+\;x^{N}}$$

where *R*(*x*) is the average response at a given contrast value, x, *b* controls the steepness of the curve, and *C*_*50*_ is the point at which the curve reaches 50% of its maximum, which is a measure of contrast sensitivity [3].

To measure size-tuning curves, we fit the average response between 0 and 0.5 s after stimulus onset with a ratio-of-Gaussians model (ROG), which provides a good fit to size-tuning curves [44,45]:

$$R(x)\;=\;\frac{G_{e}{\left[erf(\frac{x}{W_{e}})\right]}^{2}}{1+G_{i}{\left[erf(\frac{x}{W_{i}})\right]}^{2}}$$

*G*_*e*_, *G*_*i*_, *W*_*i*_, and *W*_*e*_ are the gains and widths of the excitatory center and inhibitory surround, respectively; *x* is the size of the grating in degrees; *R(x)* is the response; and *erf* is the error function. The surround suppression index (SI) was calculated as:

$$SI\;=\;\frac{R_{max}-R_{supp}}{R_{max}}$$

where R_max_ was the maximum of the modeled response and R_supp_ was the response of the model to the largest size.

### Fitting the Gamma-Peak in the LFP Data

To estimate the amplitude, peak frequency, and width of the gamma peak, we fit a Gaussian function to the relative change in gamma power between 30–100 Hz using nonlinear multidimensional minimalization with the Nelder-Mead algorithm in MATLAB:

$$P(f)\;=\;\frac{Ge^{-\frac{1}{2}{\left(\frac{f-\mu }{\sigma }\right)}^{2}}}{\sqrt{2\pi }\sigma }+b,$$

where *P*(*f*) is the power at frequency *f*, *G* determines the amplitude, *b* is an offset, *μ* is the peak frequency, and *σ* is the standard deviation.

## Supporting Information

S1 Fig The derivation of MUA and MUAt from the raw signal. (A) The unprocessed signal (filtered between 0.1 Hz–9 kHz) on a single trial. The stimulus was a full-screen, 100% contrast checkerboard of 250 ms duration presented at the time marked by “flash.” (B) We filtered the raw signal between 500 Hz and 5 kHz to limit the signal to the high frequency spike-range. We applied a spike-threshold based on the unbiased estimate of the median absolute deviation (0.6745 × the median of the absolute voltage). We used a threshold of +/-3.5 times this measure for all experimental sessions except for the size-tuning data where a value of +/-4 was used. The raster plot above the graph shows threshold crossings. The threshold detection algorithm had a dead-time of 1.5 ms and the spike-time was logged as the time of maximum absolute voltage within 0.75 ms following the threshold crossing. We denote this signal here as MUAt: the thresholded multi-unit signal. (C) To calculate the envelope of the multi-unit signal we first took the absolute value of the band-limited signal. (D) We then low-passed this signal at 200 Hz to construct MUA, a measure for the envelope of activity between 500 Hz and 5 kHz. This signal has units of microvolts, but we present normalized data throughout the manuscript. The normalization procedure is described in the Materials and Methods section. (E) MUA data from 20 checkerboard trials. The individual traces were corrected for baseline activity (-300–0 ms) and smoothed with a sliding window of 21.5 ms (20 samples) duration for graphical purposes. (F) Raster plots showing MUAt on the same 20 trials. (G) The average MUA data over the 20 trials. (H) The MUAt spike-density function, created by first binning the spike-times into bins of 1.1 ms duration and then convolving the spike-train with a 22.6 ms long Gaussian density function with a standard deviation of 3.2 ms and integral of one. The convolved spike-trains were averaged to produce the spike-density plot below with units of Hertz. The spike-density function shown here was smoothed with a sliding window of 21.5 ms duration for graphical purposes. Statistics were performed on unsmoothed data for both MUA and MUAt.
(TIF)

S2 Fig Tunings of thresholded multi-unit data (MUAt). In addition to the multi-unit envelope data presented in the main body of the paper, we also analyzed the thresholded multi-unit signal (MUAt) as described in S1 Fig. (A) Receptive field mapping of MUAt. (B) Orientation tuning. MUAt from E7 was tuned for orientation (preferred orientation = 178°, HWHH = 58°, 1-CircVar = 0.11, *p* = 0.01), but the MUAt at E6 was not tuned (*p* = 0.52). (C) Spatial frequency tuning of MUAt signals at E6 and E7. Spontaneous activity was measured in a window from -0.2 to 0 s relative to stimulus onset. (D) Contrast response function of MUAt at E7. (E) Size-tuning data from E6 and E7.
(TIF)

S3 Fig Receptive fields measured using the event-related potential of the LFP. Data in the same format as Fig 2A showing RFs measured using the root mean squared voltage of the event-related response to the checks with the method described by Yoshor et al. [11]. This technique did not yield clearly localized RFs for either E6 or E7.
(TIF)

S4 Fig Consistency of MUA signals across sessions. We measured orientation tuning in two separate sessions recorded 4 (session #1) and 6 (session #2) d after electrode implantation. The graphs show MUA data from each session. The pre-stimulus spontaneous activity has been subtracted, but the data has not been normalized to allow a comparison of signal magnitude across sessions. The red line shows responses to the preferred orientations (averaged across 165° and 180°) and the black line shows the response to the orthogonal orientations (averaged across 75° and 90°). Responses from session #2 were somewhat weaker. In our experience, the magnitude of the spiking activity tends to decrease over time, but the basic orientation-tuned response remained intact.
(TIF)

S5 Fig Contextual modulation elicited by a 4° diameter grating. Data in the same format as Fig 6B–6E. * = *p* < 0.05, ** = *p* < 0.01, *** = *p* < 0.001. In the main text we presented data using a 6° diameter grating where the edges of the grating were outside the RF. It can be seen that the results obtained with a 4° diameter grating were very similar.
(TIF)

S6 Fig Eye movement data from the curve-tracing experiment. (A) Examples of eye position traces during the curve-tracing paradigm. We rejected trials with microsaccades during the analysis period (0–500 ms after stimulus onset), which were detected based on a velocity threshold of 10°.s-1 maintained for at least 10 ms (the eye position sampling rate was 1,000 Hz). Examples of accepted and rejected trials are shown in the upper and lower panel, respectively. The long fixation period resulted in a large number of trials being excluded due to microsaccades (59.3% of all correct trials). (B) The mean eye position on correct trials in the period 0–500 ms from trials in which the curve passing through the RFs was connected to fixation (target: red dots) and trials in which the other curves were connected (distracter: blue dots). The average eye position is given by the larger circles. (C) The mean eye-traces from all trials for the *x* (left panel) and *y* (right panel) positions. The position of the eyes was very stable during the analysis period, with no differences between the conditions.
(TIF)

## Abbreviations

1-CircVar: 1 –circular variance
ECoG: electrocorticography
EEG: Electroencephalography
FGM: figure-ground modulation
fMRI: functional magnetic resonance imaging
FWHM: full width at half maximum
HWHM: half width at half maximum
LFP: local field potential
MEG: magnetoencephalography
MUA: multi-unit spiking activity
MUAt: thresholded multi-unit spiking activity
OTSS: orientation-tuned surround suppression
RF: receptive field
SI: surround index
