# Supplementary figures and images for: The Effects of Context and Attention on Spiking Activity in Human Early Visual Cortex
Source: PLoS Biol. 2016 Mar 25;14(3):e1002420. doi: 10.1371/journal.pbio.1002420 (PMC4807817; doi:10.1371/journal.pbio.1002420)

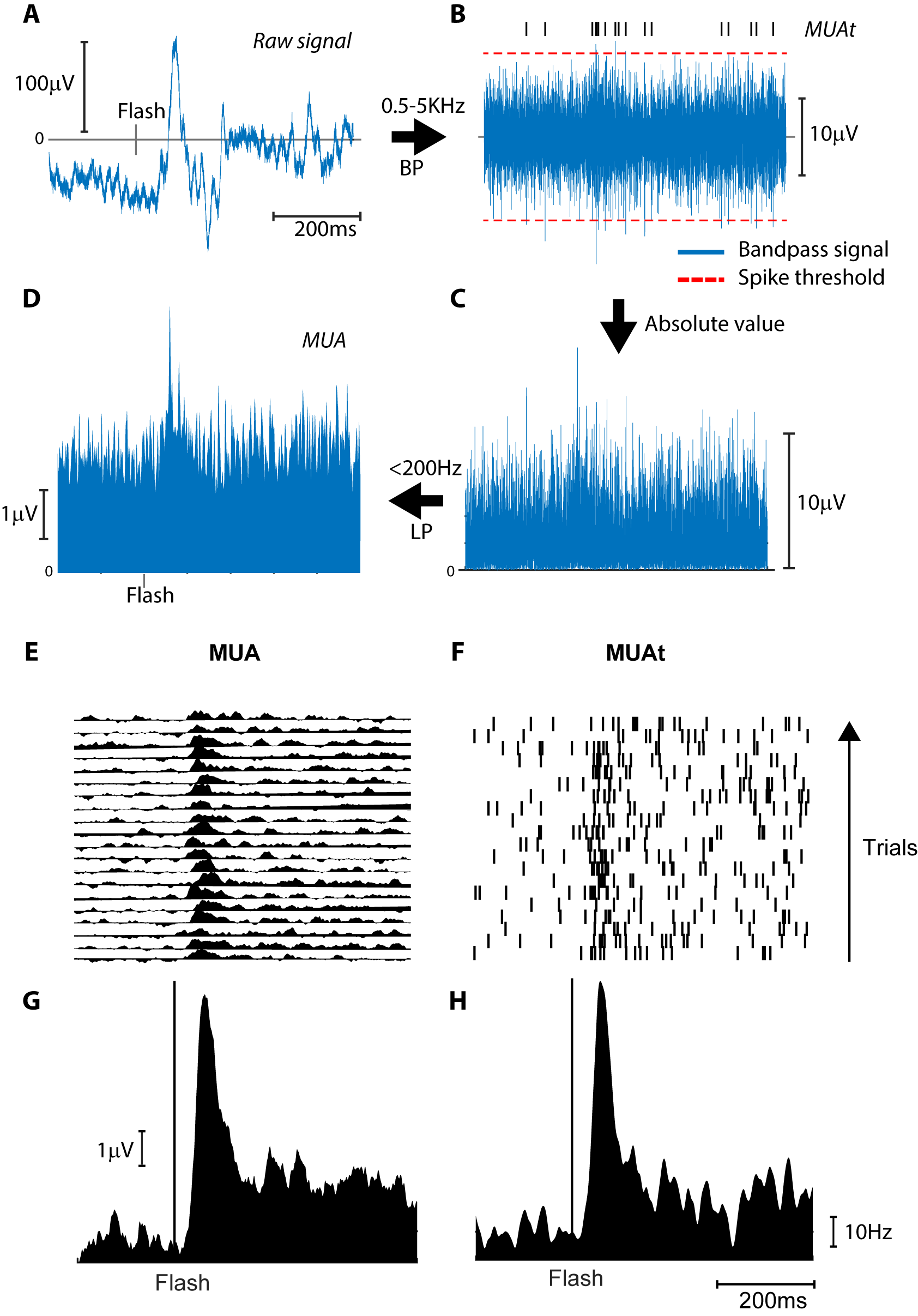

Supplement: S1 Fig — (A) The unprocessed signal (filtered between 0.1 Hz–9 kHz) on a single trial. The stimulus was a full-screen, 100% contrast checkerboard of 250 ms duration presented at the time marked by “flash.” (B) We filtered the raw signal between 500 Hz and 5 kHz to limit the signal to the high frequency spike-range. We applied a spike-threshold based on the unbiased estimate of the median absolute deviation (0.6745 × the median of the absolute voltage). We used a threshold of +/-3.5 times this measure for all experimental sessions except for the size-tuning data where a value of +/-4 was used. The raster plot above the graph shows threshold crossings. The threshold detection algorithm had a dead-time of 1.5 ms and the spike-time was logged as the time of maximum absolute voltage within 0.75 ms following the threshold crossing. We denote this signal here as MUAt: the thresholded multi-unit signal. (C) To calculate the envelope of the multi-unit signal we first took the absolute value of the band-limited signal. (D) We then low-passed this signal at 200 Hz to construct MUA, a measure for the envelope of activity between 500 Hz and 5 kHz. This signal has units of microvolts, but we present normalized data throughout the manuscript. The normalization procedure is described in the Materials and Methods section. (E) MUA data from 20 checkerboard trials. The individual traces were corrected for baseline activity (-300–0 ms) and smoothed with a sliding window of 21.5 ms (20 samples) duration for graphical purposes. (F) Raster plots showing MUAt on the same 20 trials. (G) The average MUA data over the 20 trials. (H) The MUAt spike-density function, created by first binning the spike-times into bins of 1.1 ms duration and then convolving the spike-train with a 22.6 ms long Gaussian density function with a standard deviation of 3.2 ms and integral of one. The convolved spike-trains were averaged to produce the spike-density plot below with units of Hertz. The spike-density function show [file pbio.1002420.s001.tif]

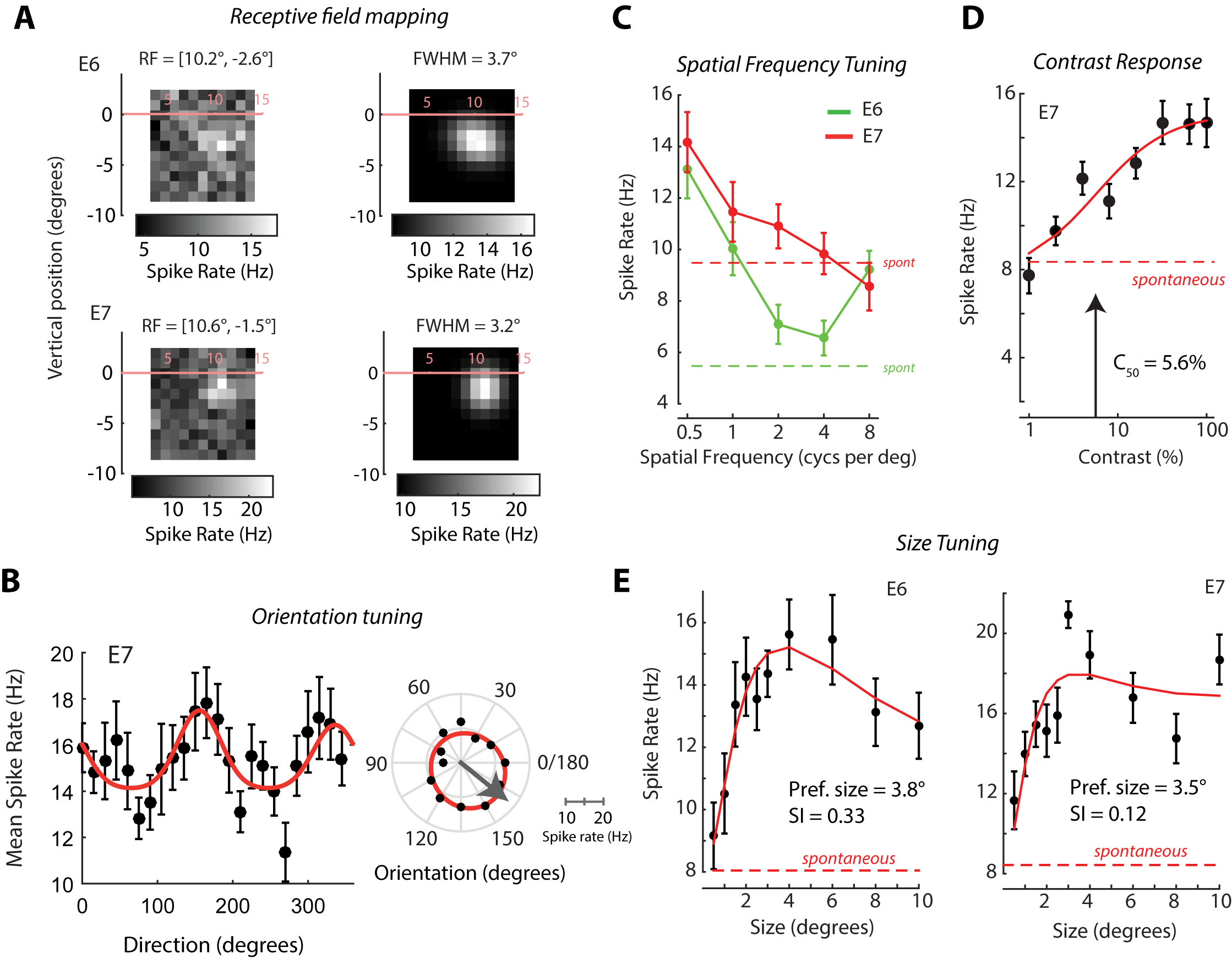

Supplement: S2 Fig — In addition to the multi-unit envelope data presented in the main body of the paper, we also analyzed the thresholded multi-unit signal (MUAt) as described in S1 Fig. (A) Receptive field mapping of MUAt. (B) Orientation tuning. MUAt from E7 was tuned for orientation (preferred orientation = 178°, HWHH = 58°, 1-CircVar = 0.11, p = 0.01), but the MUAt at E6 was not tuned (p = 0.52). (C) Spatial frequency tuning of MUAt signals at E6 and E7. Spontaneous activity was measured in a window from -0.2 to 0 s relative to stimulus onset. (D) Contrast response function of MUAt at E7. (E) Size-tuning data from E6 and E7. (TIF) [file pbio.1002420.s002.tif]

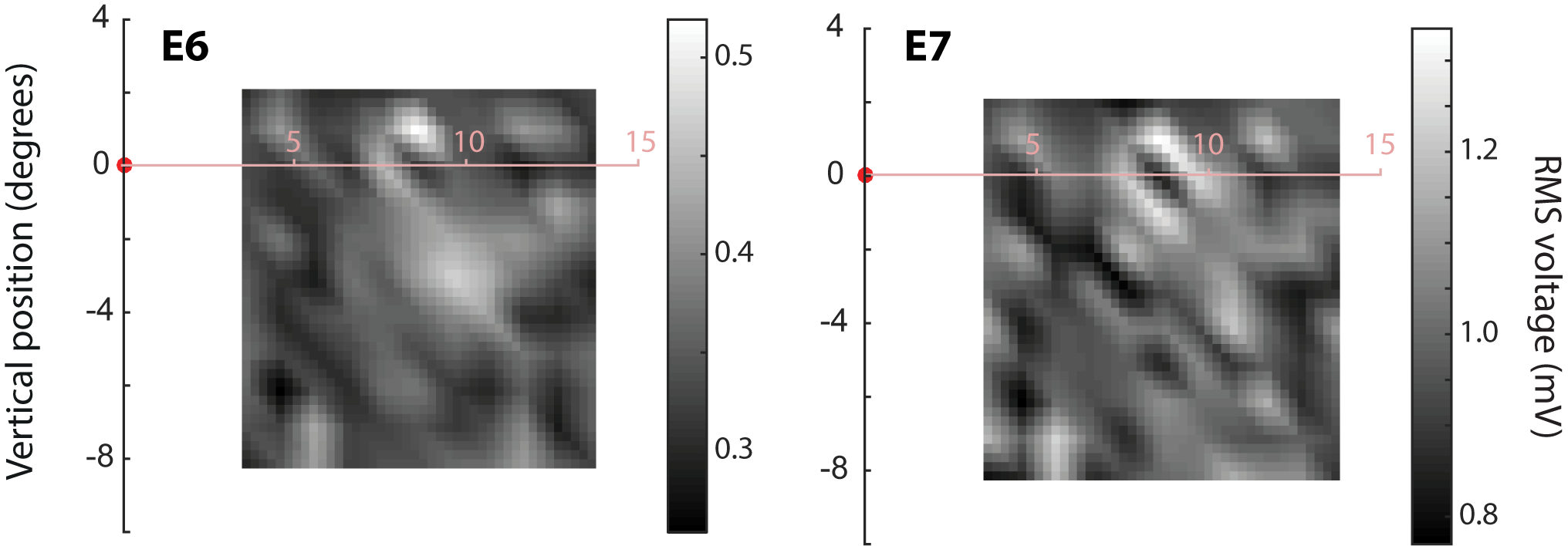

Supplement: S3 Fig — Data in the same format as Fig 2A showing RFs measured using the root mean squared voltage of the event-related response to the checks with the method described by Yoshor et al. [11]. This technique did not yield clearly localized RFs for either E6 or E7. (TIF) [file pbio.1002420.s003.tif]

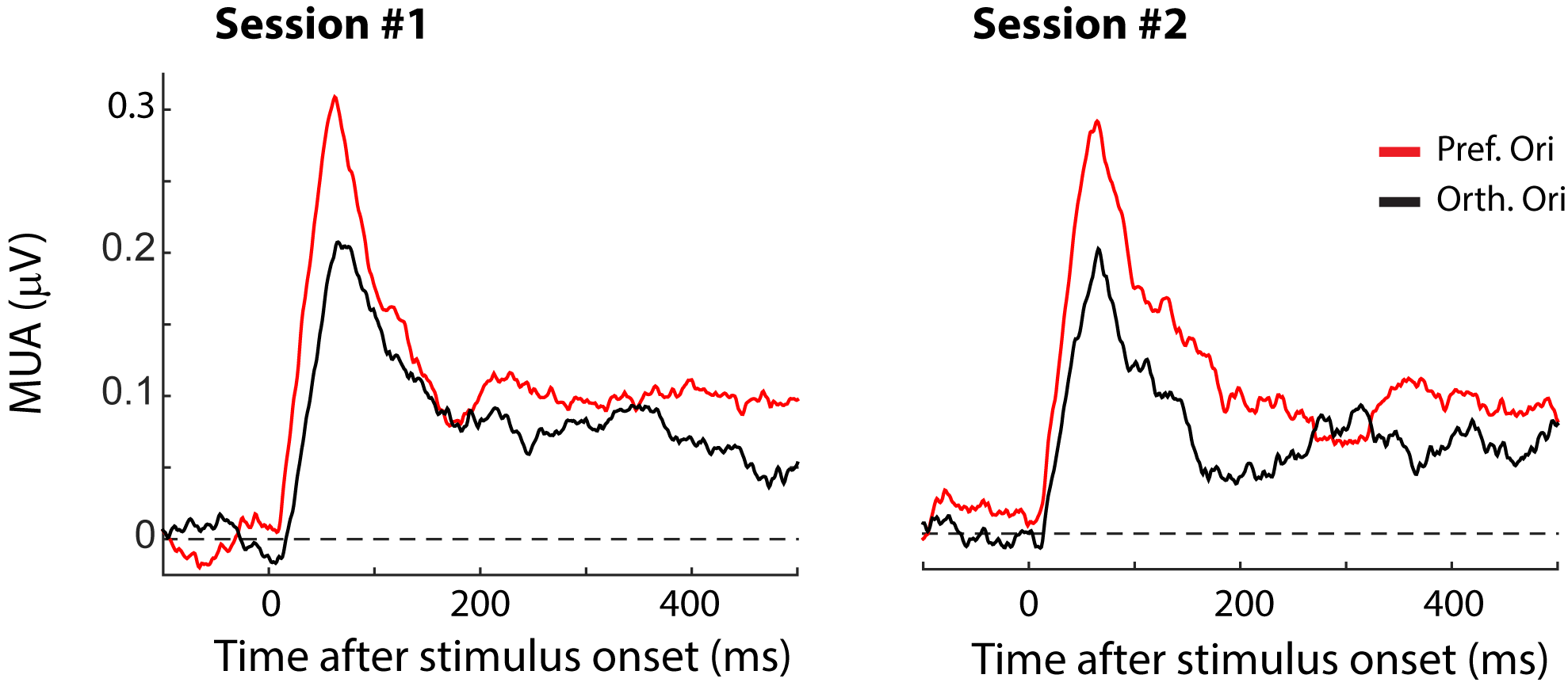

Supplement: S4 Fig — We measured orientation tuning in two separate sessions recorded 4 (session #1) and 6 (session #2) d after electrode implantation. The graphs show MUA data from each session. The pre-stimulus spontaneous activity has been subtracted, but the data has not been normalized to allow a comparison of signal magnitude across sessions. The red line shows responses to the preferred orientations (averaged across 165° and 180°) and the black line shows the response to the orthogonal orientations (averaged across 75° and 90°). Responses from session #2 were somewhat weaker. In our experience, the magnitude of the spiking activity tends to decrease over time, but the basic orientation-tuned response remained intact. (TIF) [file pbio.1002420.s004.tif]

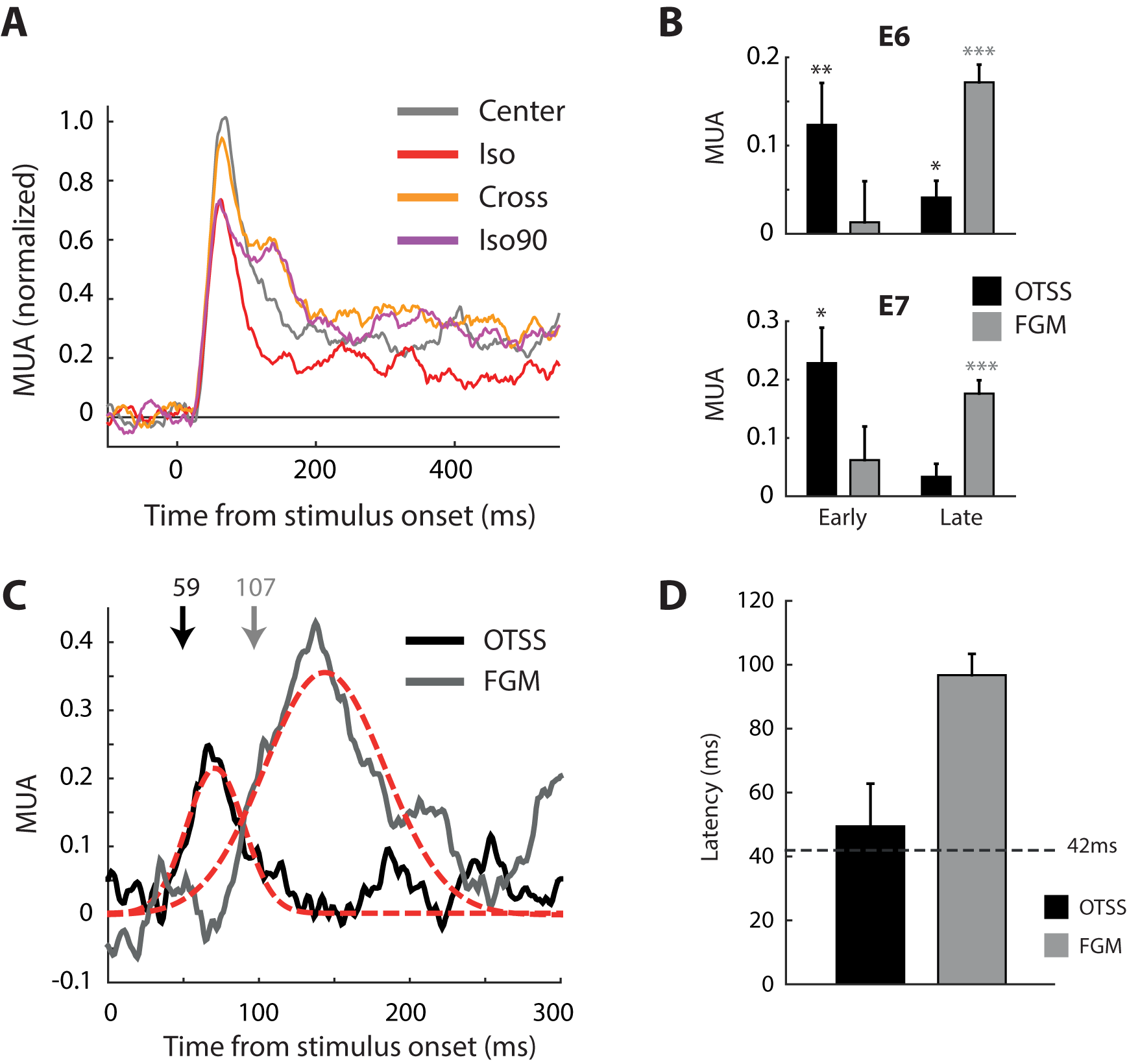

Supplement: S5 Fig — Data in the same format as Fig 6B–6E. * = p < 0.05, ** = p < 0.01, *** = p < 0.001. In the main text we presented data using a 6° diameter grating where the edges of the grating were outside the RF. It can be seen that the results obtained with a 4° diameter grating were very similar. (TIF) [file pbio.1002420.s005.tif]

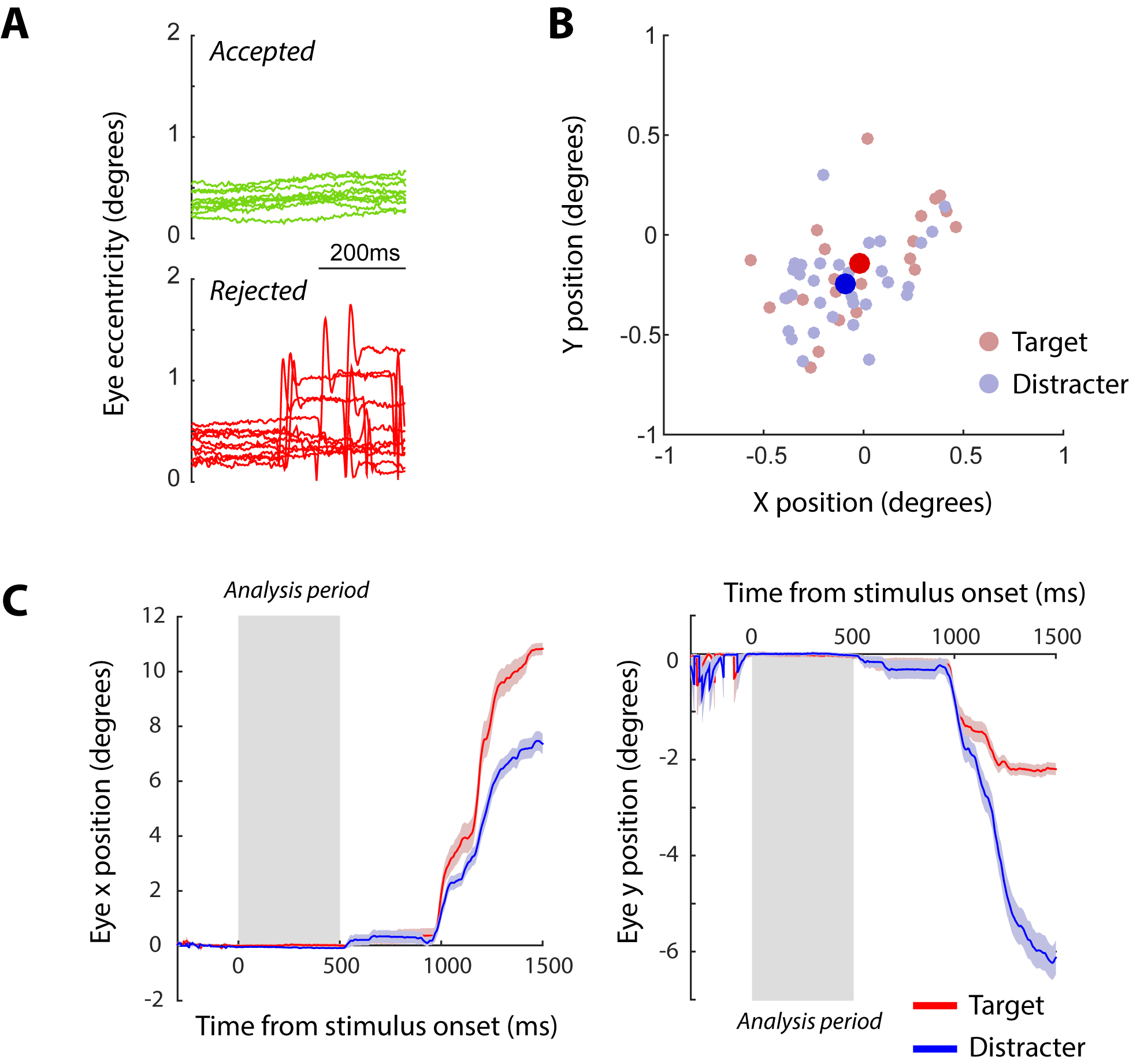

Supplement: S6 Fig — (A) Examples of eye position traces during the curve-tracing paradigm. We rejected trials with microsaccades during the analysis period (0–500 ms after stimulus onset), which were detected based on a velocity threshold of 10°.s-1 maintained for at least 10 ms (the eye position sampling rate was 1,000 Hz). Examples of accepted and rejected trials are shown in the upper and lower panel, respectively. The long fixation period resulted in a large number of trials being excluded due to microsaccades (59.3% of all correct trials). (B) The mean eye position on correct trials in the period 0–500 ms from trials in which the curve passing through the RFs was connected to fixation (target: red dots) and trials in which the other curves were connected (distracter: blue dots). The average eye position is given by the larger circles. (C) The mean eye-traces from all trials for the x (left panel) and y (right panel) positions. The position of the eyes was very stable during the analysis period, with no differences between the conditions. (TIF) [file pbio.1002420.s006.tif]
